# Sodium butyrate inhibits the expression of virulence factors in *Vibrio cholerae* by targeting ToxT protein

**DOI:** 10.1128/msphere.00824-24

**Published:** 2025-04-22

**Authors:** Sushmita Kundu, Suman Das, Priyanka Maitra, Prolay Halder, Hemanta Koley, Asish K. Mukhopadhyay, Shin-ichi Miyoshi, Shanta Dutta, Nabendu Sekhar Chatterjee, Sushmita Bhattacharya

**Affiliations:** 1 Division of Biochemistry, ICMR-National Institute for Research in Bacterial Infections (Formerly ICMR-National Institute of Cholera and Enteric Diseases) 30170 , Kolkata, India; 2 Division of Bacteriology, ICMR-National Institute for Research in Bacterial Infections (Formerly ICMR-National Institute of Cholera and Enteric Diseases) 30170 , Kolkata, India; 3 Division of Pharmaceutical Sciences, Graduate School of Medicine, Dentistry and Pharmaceutical Sciences, Okayama University 12997, Okayama, Japan; The University of Texas Medical Branch at Galveston, Galveston, Texas, USA

**Keywords:** sodium butyrate (SB), inhibitor, pathogenesis, *Vibrio cholerae*, *ctxAB*, antimicrobial resistance, toxin-coregulated pilus (TcpA)

## Abstract

Cholera, a diarrheal disease caused by the gram-negative bacterium *Vibrio cholerae*, remains a global health threat in developing countries due to its high transmissibility and increased antibiotic resistance. There is a pressing need for alternative strategies, with an emphasis on anti-virulent approaches to alter the outcome of bacterial infections, given the increase in antimicrobial-resistant strains. *V. cholerae* causes cholera by secreting virulence factors in the intestinal epithelial cells. These virulence factors facilitate bacterial colonization and cholera toxin production during infection. Here, we demonstrate that sodium butyrate (SB), a small molecule, had no effect on bacterial viability but was effective in suppressing the virulence attributes of *V. cholerae*. The production of cholera toxin (CT) was significantly reduced in a standard *V. cholerae* El Tor strain and two clinical isolates when grown in the presence of SB. Analysis of mRNA and protein levels further revealed that SB reduced the expression of the ToxT-dependent virulence genes like *tcpA* and *ctxAB*. DNA-protein interaction assays, conducted at cellular (ChIP) and *in vitro* conditions (EMSA), indicated that SB weakens the binding between ToxT and its downstream promoter DNA, likely by blocking DNA binding. Furthermore, the anti-virulence efficacy of SB was confirmed in animal models. These findings suggest that SB could be developed as an anti-virulence agent against *V. cholerae,* serving as a potential alternative to conventional antibiotics or as an adjunctive therapy to combat cholera.

**IMPORTANCE:**

The world has been facing an upsurge in cholera cases since 2021, a similar trend continuing into 2022, with over 29 countries reporting cholera outbreaks (World Health Organization, 16 December 2022, Disease Outbreak News, Cholera—global situation). Treatment of cholera involves oral rehydration therapy coupled with antibiotics to reduce the duration of the illness. However, in recent years, indiscriminate use of antibiotics has contributed to the emergence of antibiotic-resistant strains. In this study, we have addressed the problem of antibiotic resistance by targeting virulence factors. Screening various compounds using *in silico* methods led to the identification of a small molecule, SB, that inhibits the virulence cascade in *V. cholerae*. We demonstrated that (i) SB intervened in ToxT protein-DNA binding and subsequently affected the expression of ToxT-regulated virulence genes (*ctxAB* and *tcpA*) and (ii) SB is a potential therapeutic candidate for the development of a novel antimicrobial agent.

## INTRODUCTION

Cholera is an acute, severely dehydrating diarrheal disease caused by water-borne bacterium *Vibrio cholerae* (1). Since mid-2021, the world has witnessed a drastic upsurge of the ongoing seventh cholera pandemic, characterized by frequent outbreaks along with the increased incidence mortality rate (1.9%) (2). This trend continued into 2022, and as of February 2023, at least 18 countries continued reporting cholera cases. The mortality linked to these outbreaks was particularly concerning, with many countries reporting higher case fatality ratios (CFR) compared with previous years (2). Although over 200 *V*. *cholerae* serogroups have been identified, only the O1 (classical and El Tor biotypes) and O139 serogroups of *V. cholerae* have been implicated in epidemic and pandemic cholera (3, 4).

The clinical symptoms of cholera are primarily driven by two essential virulence factors, cholera toxin (CT) and toxin-coregulated pilus (TCP) (5). CT is an A-B_5_ family toxin directly responsible for inducing profuse watery cholera diarrhea (6, 7), whereas TCP is required for intestinal colonization (8). Coordinated expression of these virulence genes is directly under the control of the master regulator, ToxT, and given its pivotal role, numerous studies have been focused on its regulation, leading to the characterization of multiple mechanisms that contribute to its stringent regulation. ToxT belongs to the AraC/XylS family of transcriptional activators and consists of two domains: an N-terminal domain that has been linked to effector binding and potential ToxT monomer association, and a C-terminal DNA-binding domain that contains AraC/XylS homology (9). The transcription of *toxT* is regulated by two membrane-localized complexes ToxRS and TcpPH (10). TcpPH is further activated by two activators, AphA and AphB, which respond to cell density, anaerobiosis, and other environmental factors (11, 12).

The primary therapy of cholera is an oral rehydration solution (ORS), which contains different types of salts and glucose, to avoid dehydration (13, 14). Without intervention, the survival rate for cholera can be as low as 50%; however, ORS supplementation reverses the survival rate to more than 99% (15). Antibiotics are a secondary treatment in severe cases to shorten the duration of the illness (16). Although antibiotics can effectively reduce cholera burden, WHO does not recommend this practice due to the risk of developing and spreading drug-resistant bacteria (17–19). There are also vaccines against *V. cholerae,* but their efficacy is not 100% (20). Consequently, there is an unmet need for clinical intervention to control the spread of drug-resistant bacteria through rapid preventive measures.

Anti-virulence drugs are gaining popularity as an alternative approach to combat bacterial infections. Unlike antibiotics, these drugs disarm the pathogen by targeting its virulence factors and further activate the immune system to eradicate the infection (21). This strategy exerts less selective pressure on the emergence of resistant strains and reduces the impact on commensal microbiota. Previous studies identified small molecules such as toxtazin B, unsaturated fatty acids, and ribavirin targeting the virulence gene regulatory cascade (15, 22, 23). Along with small molecules, several herbal products and bioactive compounds are also reported to be potent repressors of the virulence factors, such as anethole (24), capsaicin (25) inhibiting the CT production, zinc oxide nanoparticles disrupting the secondary structure of CT (26), carbohydrate inhibitors (27), and fucosylated molecules (28), interfering with the activity of CT.

Short-chain fatty acids (SCFA), such as butyrate, are microbial metabolites synthesized from the fermentation of dietary fibers in the colonic lumen. Multiple studies have documented the substantial effects of butyrate on host immunity, energy metabolism, and overall health (29). In colorectal cells, butyrate treatment is reported to induce the production of antimicrobial peptide, cathelicidin (30). The anti-microbial and anti-virulence activity of butyrate has been well characterized in a variety of pathogenic bacteria such as *Salmonella* Typhimurium, *Clostridium perfringens* (31), *Staphylococcus pseudointermedius*, *Acinetobacter baumannii* (32), *Vibrio campbellii* (33), and *Vibrio parahaemolyticus* (34). Despite extensive research on butyrate, its antimicrobial activity against *V. cholerae* has not been reported.

In this study, we aimed to screen potential bioactive compounds against *V. cholerae* and understand the mechanism behind antivirulence activities against standard and resistant strains.

## RESULTS

### *In silico* screening of bioactive compounds identifies SB as a potential inhibitor

**Fig 1:**
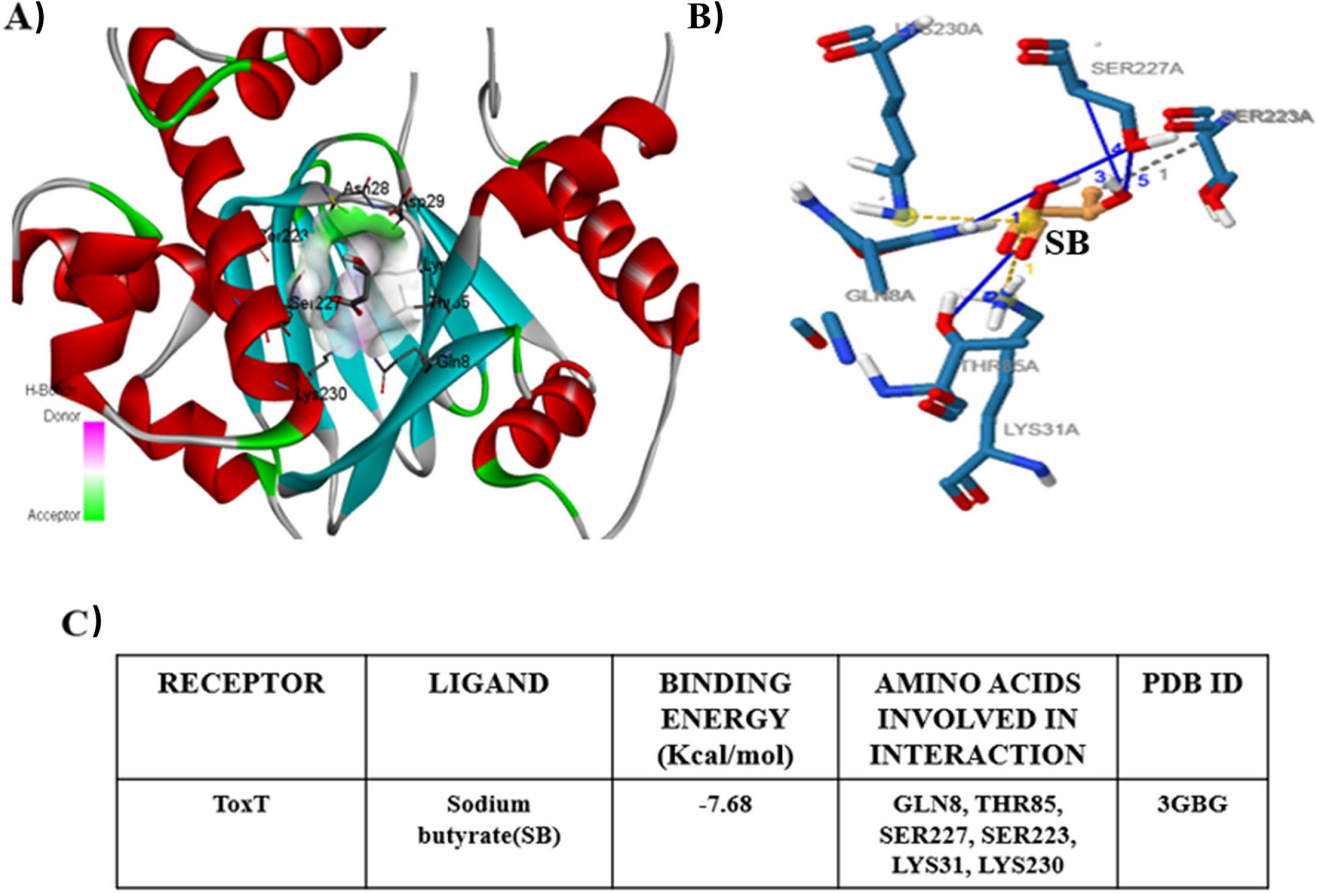
Binding of SB with ToxT *in silico*. Residues interacting with ligand (SB) and receptor (ToxT) are represented in (A) 3D conformation, (B) 2D conformation, and (C) table showing the probable interacting residues and the binding energy.

Among different transcriptional activators present in *V. cholerae*, targeting ToxT has frequently been reported to have additional advantages for developing new antimicrobial drugs, aimed at targeting the virulence factor rather than bacterial growth. Hence, to identify potential inhibitors, we have used an *in silico* virtual screening technique, important for the drug discovery process. To focus on potent ToxT inhibitors, several bioactive compounds (Table S1) were docked individually with the binding pocket of ToxT protein (PDB ID: 3GBG). The possible amino acid residues present in the binding pocket of ToxT are Ile226, Tyr26, Asn28, Asp29, Ser223, Ser227, Asn60, Thr85, Gln8, Lys31, and Lys230. Based on the docking pose and possible interaction, the binding energy of the compounds is listed in Table S1. Among the docked ligands, the compound SB showed the highest binding affinity of −7.68 Kcal/mol and stable interaction with ToxT, forming two salt bridges with Lys31 and Lys230 and a single hydrogen bond with Lys31 (Fig. 1A and B). Other residues involved in interactions with SB are summarized in Fig. 1C. Altogether, these data suggest that SB may interact with ToxT and therefore are chosen for subsequent *in vitro* and *in vivo* experiments to test their efficacy against the virulence factors in *V. cholerae*.

### The viability of bacterial strains and cytotoxicity in the HT-29 cell line in the presence of SB

**Fig 2:**
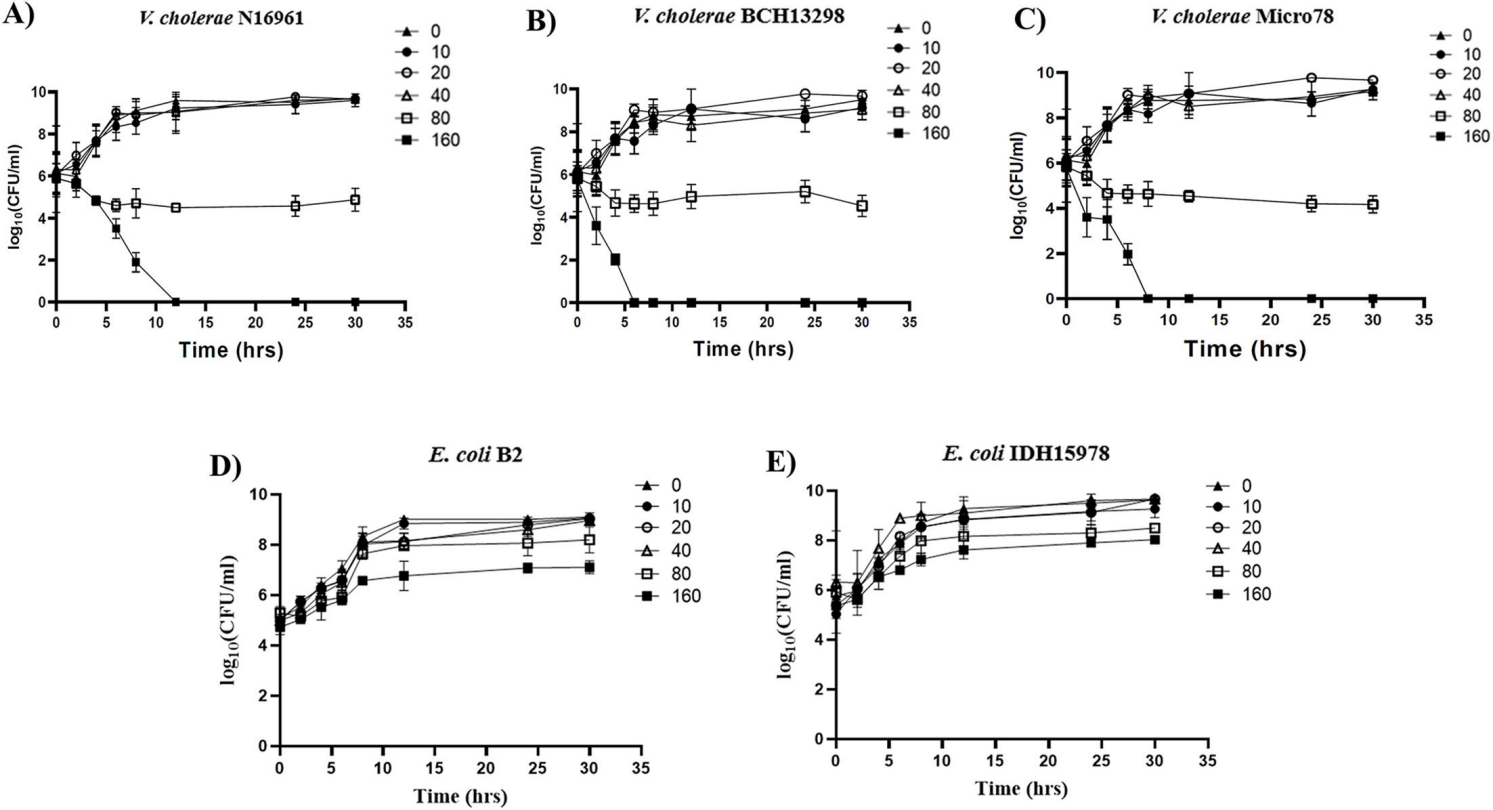
Effect of SB on the bacterial growth at MIC and sub-MICs. El Tor *V. cholerae* strains were grown in LB broth along with SB at MBC (160 mM), SB 160 (■); MIC (80 mM), SB 80 (□); 1/2^th^ MIC (40 mM), SB 40 (Δ), 1/4^th^ MIC (20 mM), SB 20 (○), 1/8^th^ MIC (10 mM), SB 10 (●), and SB 0 (▲) for the indicated periods (2, 4, 6, 8, 12, 24, and 30 h). The viable bacterial counts in CFU per mL detected by the plate count method were represented graphically for (A) multidrug-resistant *V. cholerae* N16961, (B) multidrug-resistant *V. cholerae* BCH13298, (C) multidrug-resistant *V. cholerae* Micro78, (D) multidrug-resistant *E. coli* B2, and (E) multidrug-resistant *E. coli* IDH15978.

To evaluate the antibacterial activity of SB, the MIC and MBC values of SB against bacterial strains were determined (Fig. S1A). The growth rate of the strains in the presence of SB was evaluated in a time-dependent manner (Fig. 2). The results indicated complete bactericidal activity of SB against N16961 at 160 mM (MBC), as no live bacteria could be detected after 12 h (Fig. 2A). Notably, the bactericidal activity of SB (160 mM) observed against the other two strains was more potent than N16961, as no live bacteria could be detected after 6 h in BCH13298 and after 8 h in Micro78 (Fig. 2B and C). At 80 mM (MIC) of SB, the growth pattern of all the bacterial strains was similar, with a significant reduction observed in viability count compared with the untreated *V. cholerae* cells (Fig. 2A through C). At 10–40 mM (sub-MICs) of SB, there was no significant difference in growth rate as compared with the untreated cells (Fig. 2A through C). Additionally, we tested whether SB exhibits antibacterial activities against *E. coli* strains. The results, as shown in Fig. 2D and E, indicate that the antibacterial effect of SB on *E. coli* B2 and *E. coli* IDH15978 was not as pronounced as that observed for *V. cholerae* strains. Even at the highest concentration of SB tested (=160 mM), viable colonies of *E. coli* strains could still be detected after 30 h, emphasizing *V. cholerae*-specific bactericidal activity of SB. The sub-MICs of SB were used in all subsequent *in vitro* experiments. *In vivo* experiments were performed at both sub-MICs and MIC of SB to compare the effect with the different groups.

Toxicity of SB in mammalian cell lines was also evaluated. No significant cytotoxicity was observed when HT-29 cells were incubated with varying doses of SB (5–160mM) (Fig. S1B).

### SB inhibits CT and TcpA production

**Fig 3:**
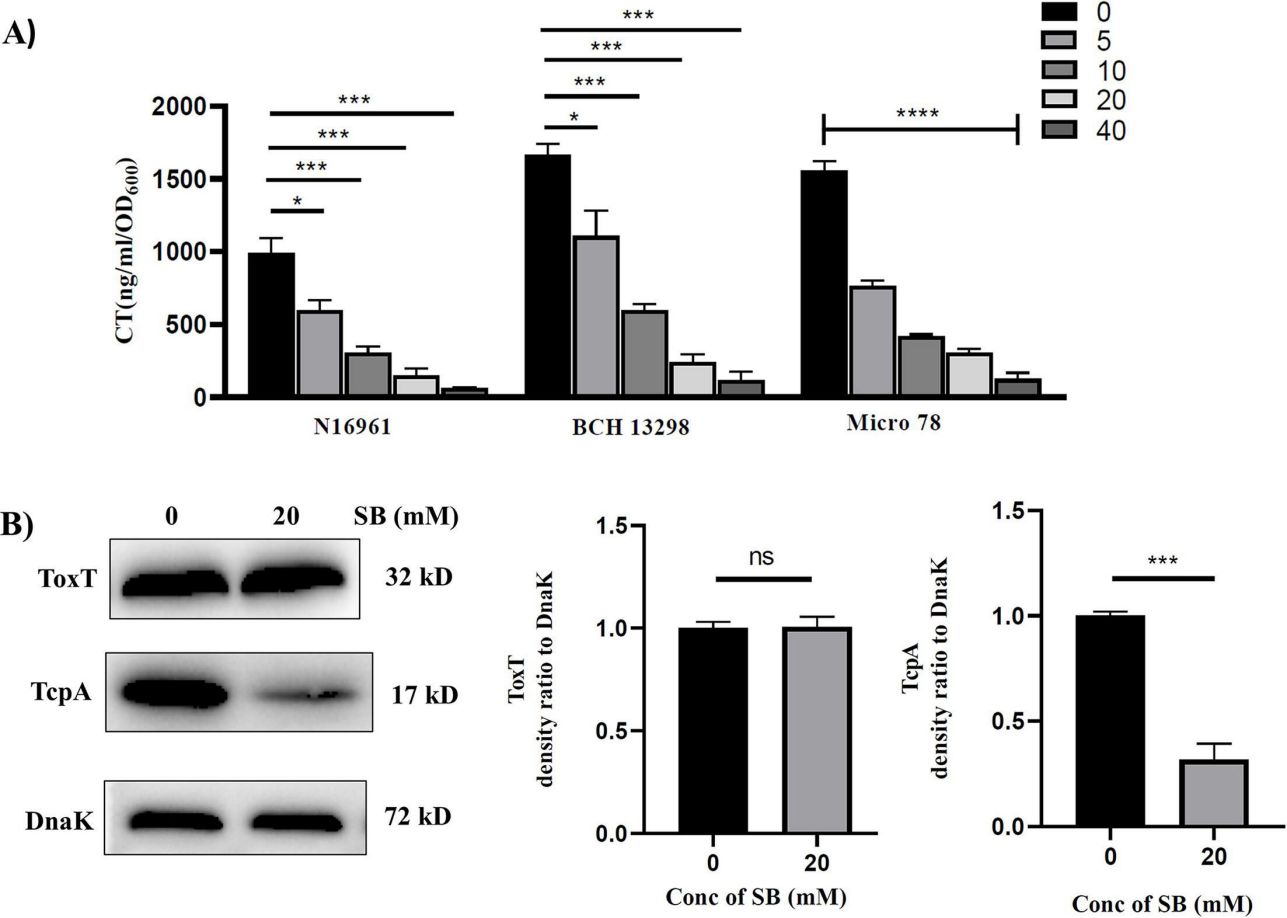
Inhibitory effects of SB on the virulence attributes of *V. cholerae*. (A) CT expression levels in different O1 El Tor strains were measured by ELISA from the samples grown in AKI media with or without SB. One-way ANOVA was performed. (B) Expression of major virulence proteins ToxT and TcpA of *V. cholerae* N16961 measured by western blot of cells grown in the presence or absence of SB (20 mM). DnaK was used as a loading control. Densitometric analyses are graphically represented. Student’s *t*-test was performed. All data are expressed as mean ± S.D. from three biological replicates. Significance levels were denoted as ∗ for *P* < 0.05, ∗∗∗ for *P* < 0.001, ∗∗∗∗ for *P* < 0.0001.

The above *in silico* results predict that SB forms stable interaction with ToxT. Given the role of ToxT in regulating *V. cholerae* virulence genes, we sought to investigate whether SB could affect the expression of ToxT-regulated virulence genes. First, we explored the classical GM1-CT ELISA for detecting the secreted CT level in the supernatant of bacterial cultures grown in the presence or absence of sub-MICs of SB (10–40 mM). It was observed that SB significantly inhibited CT production in all three *V. cholerae* strains in a dose-dependent manner (Fig. 3A). Since the expression of CT is coordinately regulated with the expression of TcpA, N16961 cultures grown in the presence of SB were also analyzed for TcpA expression by immunoblot. SB significantly decreased the TcpA levels relative to the untreated cells (Fig. 3B).

To understand the underlying mechanism behind the reduced production of CT and TcpA, qRT-PCR was used to measure the expression levels of cholera toxin-encoding *ctxAB* and pilus-encoding *tcpA* genes in N16961. The expressions of *ctxAB* and *tcpA* were significantly downregulated by SB (more than 3-fold) compared with the untreated cells (Fig. S2A). In addition to this, we also determined whether SB affects the expression of genes encoding virulence regulators (i.e., *toxS, toxR, tcpH, tcpP,* and *toxT* genes) in N16961 using qRT-PCR. Notably, SB did not affect the expression of any of these genes compared with the untreated cells (Fig. S2A). To rule out the possibility that the reduced *ctxAB* and *tcpA* transcription depends on the posttranscriptional or translational regulation of *toxT*, the levels of ToxT protein were measured in the presence or absence of SB. As shown in , SB did not affect the cellular levels of the ToxT protein, indicating that SB affects the virulence cascade without affecting ToxT expression.

Adhering ability of *V. cholerae* to intestinal epithelial cells is highly dependent on TcpA. Since SB decreased the TcpA levels, we also examined the effect of SB on the adhering ability of *V. cholerae* to intestinal epithelial cells, HT-29 cells (Fig. S2B). The cell adherence assays revealed that the adhering ability of *V. cholerae* to HT-29 cells was significantly reduced in the presence of SB compared with the untreated condition (Fig. S2B).

### SB binds to the ToxT protein and affects its interaction with the target promoter DNAs

**Fig 4:**
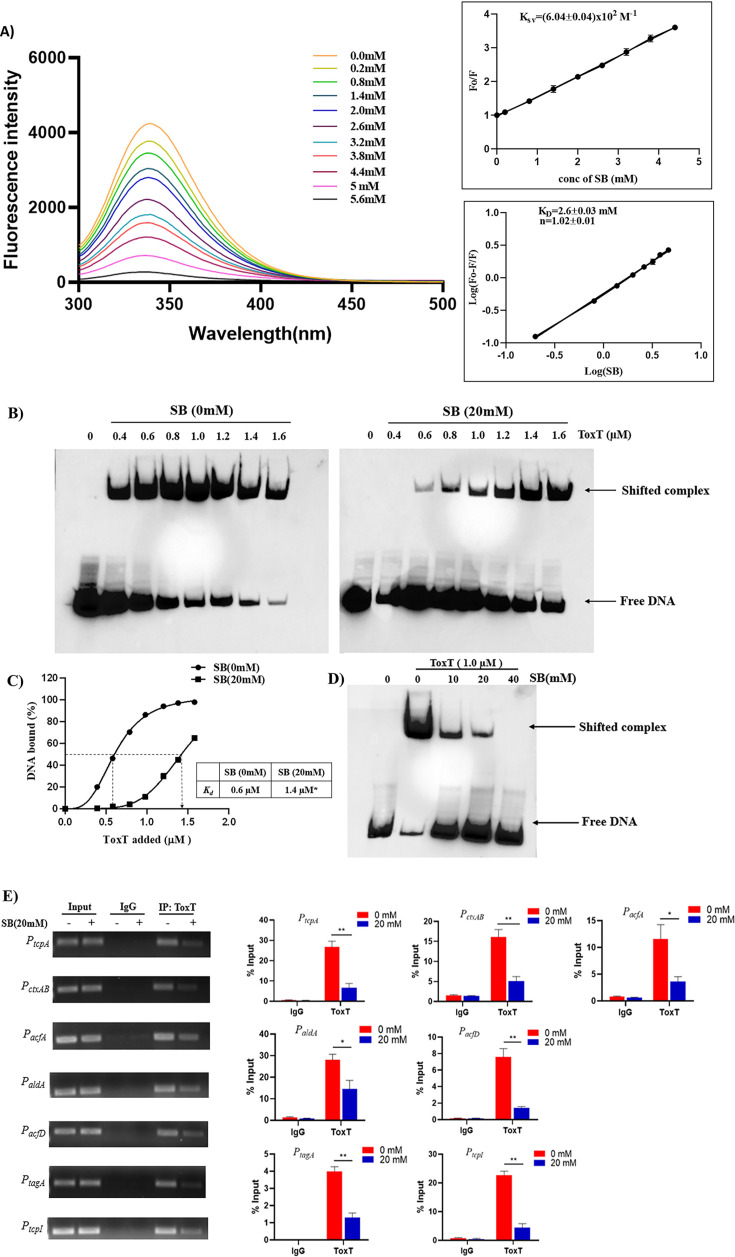
SB interacts with and inactivates the ToxT protein. (**A**) Fluorescence quenching spectra of ToxT in the presence of SB. A concentration of 0.1 µM ToxT protein was excited at 280 nm, and fluorescence quenching was recorded in the presence of various concentrations of SB (0.2–5.6 mM). Stern-Volmer plots of the decrease in fluorescence of ToxT in the presence of various concentrations of SB were used to determine the quenching rate constant, *K*_sv_ (calculated from the slope of the line). Logarithmic plots of relative fluorescence quenching of ToxT against logarithmic concentrations of SB were used to determine *K*_*D*_ (calculated from the intersection of the line with the *y*-axis) and the number of binding sites, *n* (calculated from the slope of the line). Error bars indicate standard deviations calculated from three individual experiments. (**B**) For EMSA, a 150 bp *P_tcpA_* DNA fragment was biotin-labeled and then used as a DNA probe. Purified ToxT protein (0.4, 0.6, 0.8, 1.0, 1.2, 1.4, and 1.6 µM) was pre-incubated with SB (20 mM) for 20 min, followed by incubation with the probe (1 nM) for an additional 20 min. (**C**) The relative affinities of ToxT protein in the presence or absence of SB were compared using the data from panel B. The percentage of bound DNA was calculated and plotted against the concentration of ToxT added. The *K_d_* is shown in the inset. A significant difference between the best-fit values is indicated by an asterisk (*P < 0.05). (**D**) EMSA was performed as described for panel B, except that ToxT (1.0 µM) was mixed with increasing amounts of SB (10, 20, and 40 mM). The EMSAs presented are representative of three independent experiments. (**E**) The interaction between ToxT and promoter DNAs at the cellular level in the presence or absence of SB (20 mM) was analyzed by ChIP. Expression of promoter DNAs was checked and compared to input in real-time PCR assay and agarose gel electrophoresis. Two-way ANOVA was performed. The data are expressed as mean ± S.D. (*n* = 3). IP, Immunoprecipitation; significance levels were denoted as ∗ for *P <* 0.05 and ∗∗ for *P <* 0.01.

ToxT is a direct activator of *tcpA* and *ctxAB* genes. The probable explanation for SB-mediated decrease in *tcpA* and *ctxAB* levels may be that SB can interact with and inactivate ToxT. We, therefore, examined the direct binding of SB to ToxT protein using fluorescence quenching experiment (Fig. 4A). Our results show that SB does interact with ToxT protein (*K*_sv_=[6.04 ± 0.04] × 10^2^ M^−1^ and *K*_*D*_ = 2.6 ± 0.03 mM), further supporting our *in silico* predictions.

Next, we checked whether SB could prevent the binding of ToxT to its DNA binding site, located upstream of the *tcpA* gene. Hence, we performed an electrophoretic mobility shift assay (EMSA). The EMSA results revealed the binding of ToxT to the *P_tcpA_* region in a concentration-dependent manner (Fig. 4B). The interaction between ToxT and *P_tcpA_* is specific, as CytR could not interact with the *P_tcpA_* (Fig. S3B). Similarly, a 70-fold molar excess of nonspecific competitor DNA did not affect the ToxT-*P_tcpA_* binding. In contrast, a 70-fold molar excess of specific competitor DNA completely inhibited the binding, indicating the specificity of the ToxT-*P_tcpA_* interaction (Fig. S3B). Next, the effect of SB on ToxT-*P_tcpA_* binding was examined. The addition of 20 mM of SB prevented ToxT from binding to DNA (Fig. 4B and D), and the interaction was completely inhibited at 40 mM of SB (Fig. 4D). Based on the concentration of ToxT required to bind 50% of the *P_tcpA_*, the *K_d_* for ToxT without SB was 0.6 µM, whereas that with 20 mM SB was 1.4  µM (Fig. 4C). The increase in *K_d_* value in the presence of SB further strengthens the fact that SB significantly affected the equilibrium between DNA-bound ToxT and free DNA (Fig. 4C). Another butyrate derivative, tributyrate (TB), was used, and it showed no inhibition on ToxT-DNA binding (Fig. S3C). We also checked the alternative hypothesis that SB can interact with DNA and inhibit the DNA-protein binding. As shown in Fig. S3D, no inhibition on DNA binding activity was observed under this reaction condition (SB incubated with DNA first instead of ToxT), indicating that SB specifically binds to ToxT but not DNA.

The results obtained from the EMSAs indicated that SB affects the DNA binding ability of ToxT *in vitro*. Similarly, we performed a chromatin immunoprecipitation (ChIP) assay to determine whether SB could affect the ToxT-DNA binding under physiological conditions where ToxT co-exist with other transcriptional factors. As predicted, ToxT occupancy at the *tcpA*(*P_tcpA_*) and *ctxAB*(*P_ctxAB_*) promoter region was drastically reduced in SB-treated *V. cholerae* cells compared with untreated cells, suggesting a strong interference of SB on the ToxT-*P_tcpA_* and ToxT-*P_ctxAB_* binding (Fig. 4E). In addition to this, we also assessed whether ToxT interaction with the promoter DNAs of *acfA* (*P_acfA_*), *aldA* (*P_aldA_*), *acfD* (*P_acfD_*), *tagA* (*P_tagA_*), and *tcpI* (*P_tcpI_*) genes (ToxT-regulated genes that encode accessory virulence factors) was affected by the addition of SB. Our results indicated that ToxT occupancy at each of these promoters was also reduced in SB-treated *V. cholerae* cells compared with the untreated cells (Fig. 4E). Altogether, these data indicate that SB strongly inhibits the binding of ToxT to its various downstream promoter DNAs.

### SB triggers global changes in the transcriptome of *V. cholerae*

**Fig 5:**
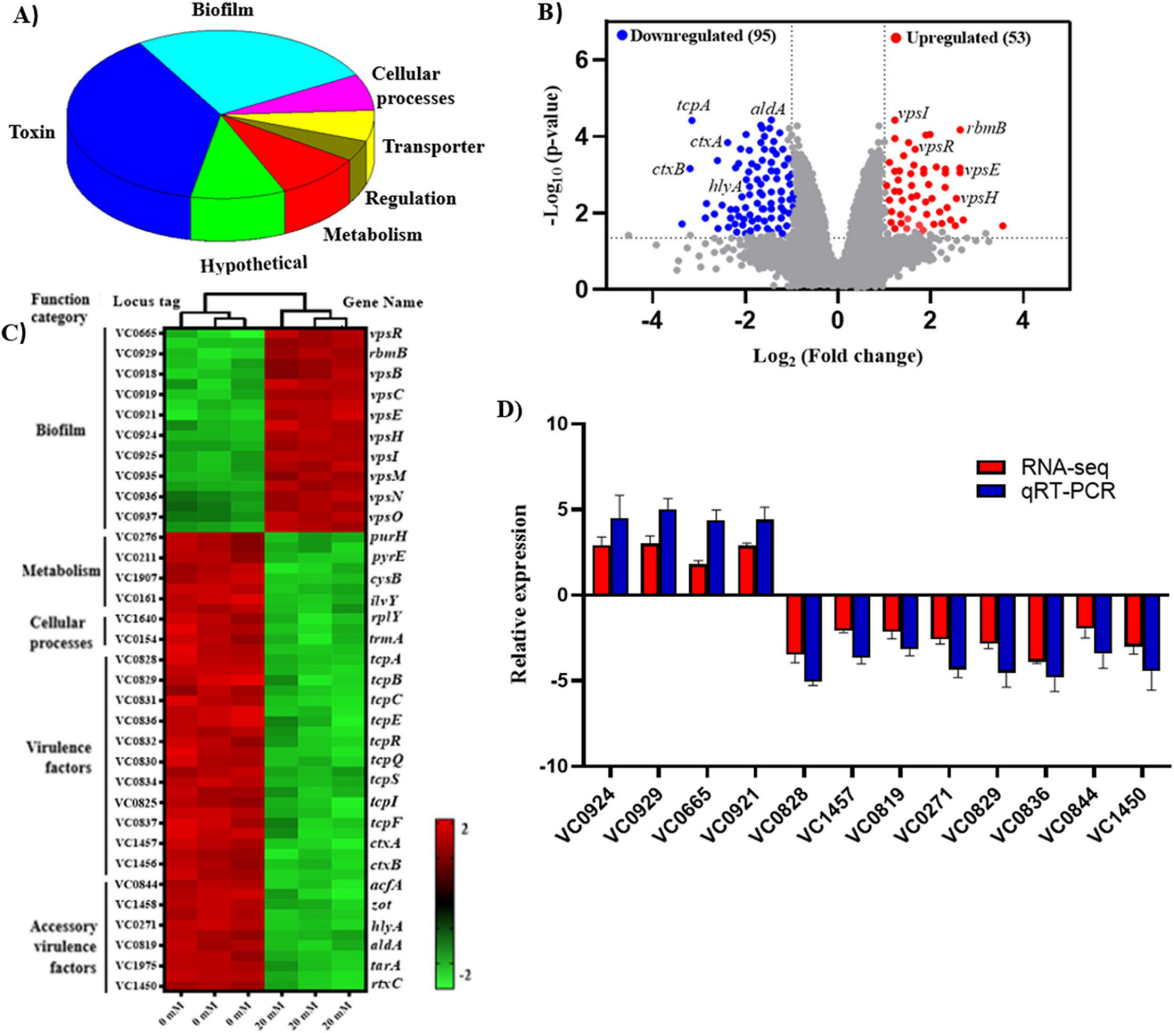
Global transcriptional responses to SB. (**A**) Genes whose expression is dysregulated in SB-treated cells (20 mM) relative to the untreated cells, as identified by RNA-seq analysis. The genes in each functional category are shown as percentages of the total genes dysregulated. (**B**) Volcano plot of differentially expressed genes of N16961 cells in the presence or absence of SB treatment. The log_2_ fold change difference is represented on the *X*-axis and −log_10_ (*P*-value) is on the *Y*-axis. Red and blue dots show upregulated and downregulated genes. (**C**) Heatmap of differentially expressed genes of N16961 cells in the presence or absence of 20 mM of SB. The z-score indicates whether the genes were upregulated (red) or downregulated (green). Columns represent independent RNA samples. (**D**) RNA-seq results validation by qRT-PCR. Data are presented as mean  ±  SD (*n*  =  3).

Next, we determined the global effects of SB on the transcriptional profile of *V. cholerae.* By using an RNA-seq approach*,* we found out that SB triggered global changes in gene expression, significantly upregulating 53 genes and downregulating 95 genes compared with untreated conditions (Fig. 5A through C). Genes primarily involved in pathogenesis, metabolism, and transcriptional/translational regulatory factors exhibited downregulation while biofilm-related genes showed upregulation (Fig. 5A through C). Twelve differentially expressed genes were randomly selected for qRT-PCR to validate the RNA-seq data. Our results showed that the expression trends of the twelve genes were consistent between RNA-seq and qRT-PCR data, suggesting that RNA-seq was reliable in identifying transcriptional changes (Fig. 5D).

### SB displays a substantial reduction in virulence attributes of *V. cholerae* in animal models

The results described so far indicate that SB has a strong negative effect on ToxT activity *in vitro*, as assessed by both gene expression (CT and TcpA production) and DNA binding experiments (EMSA). The next step was to determine whether the administration of SB in animal models for cholera would reduce virulence factor production. First, we assessed the effect of SB on the colonizing ability of *V. cholerae* N16961 in suckling mice. Intestinal colonization of N16961 in SB-treated mice (40 and 80 mM of SB administered orogastrically) was significantly reduced by at least two orders of magnitude compared with the untreated mice group (Fig. S4A). *In vivo* adherence study performed in rabbit ileal loop also revealed a significant reduction in bacterial adhesion (adherence index) in SB-treated loops compared with the untreated loop (Fig. S4B).

**Fig 6:**
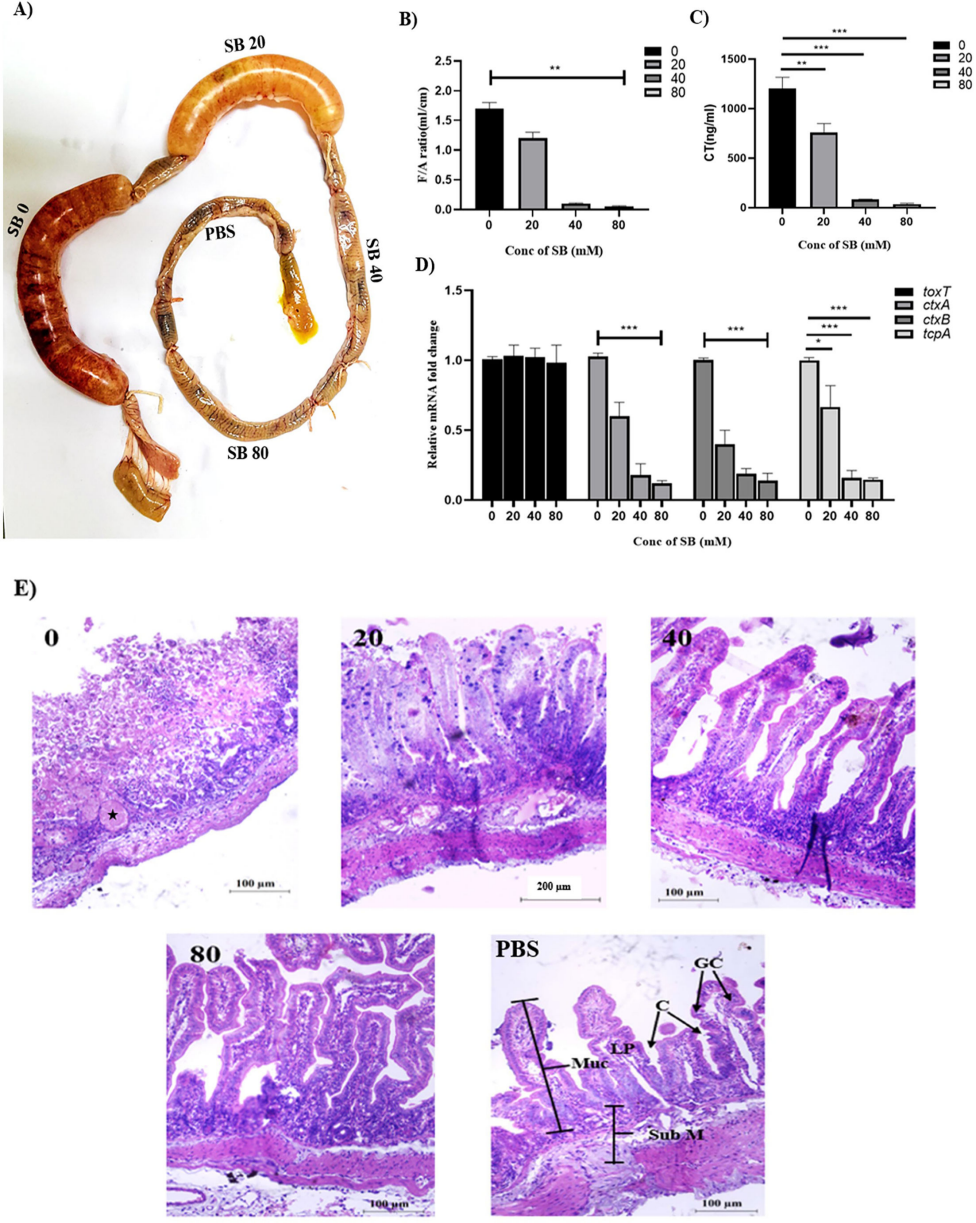
The efficacy of SB in an *in vivo* rabbit ileal loop model infected with N16961. Each loop was infected with 10^9^ CFU per mL *V. cholerae* N16961 in the presence or absence of SB (20, 40, and 80 mM). After 18 h, the animals were euthanized, and the loops were removed. (**A**) The image of the recovered rabbit intestine segment presented here is representative of three independent experiments. (**B**) The loop length and the amount of fluid accumulated in each loop were measured, and the amount of fluid (mL) per unit length (cm) of the loop was determined. (**C**) CT ELISA of rabbit ileal loop fluid produced in the presence and absence of SB. (**D**) Relative expression of major virulence genes *ctxA*, *ctxB*, *tcpA*, and *toxT* was analyzed by real-time PCR. All the data are expressed as mean ± SD from three independent experiments. Significance was calculated by one-way ANOVA. (**E**) Representative H&E staining section of rabbit intestinal tissues. In the infected loop, the damage was observed in the mucosa, submucosa, and lamina propria, along with disrupted villi and hemorrhage at the site of the muscularis mucosa. The ileal loops treated with SB showed normal microvilli structure with no alterations in the villi or mucosal structure, and no substantial damage was observed either in the submucosa or muscularis mucosa. GC, goblet cells; C, crypts; LP, lamina propria; Muc, mucosa; SubM, submucosa; *, indicating the site of hemorrhage.

The effects of SB on fluid accumulation (FA) and CT production by N16961 were also assessed in the rabbit ileal loop model (Fig. 6). FA (fluid accumulation) ratio of SB treatment loops (40 and 80 mM) showed more than 20-fold reduction in fluid accumulation compared with the loop without SB (Fig. 6B). At the two highest SB concentrations (40 and 80 mM), the CT level was markedly reduced by more than 10-fold compared with the loop that received no SB (Fig. 6C). The expression of virulence genes (*ctxAB* and *tcpA*) was downregulated in *V. cholerae* cells collected from SB-treated loop fluid compared to the untreated loop (Fig. 6D).

We also checked the *V. cholerae* virulence in the presence of TB. As anticipated, the administration of TB to suckling mice did not reduce intestinal colonization of N16961 compared with untreated mice (Fig. S4A). Similarly, no reduction in fluid accumulation and CT level was observed in the TB-treated ileal loop compared with the untreated loop (Fig. S4D through F). The expression profile of virulence genes of *V. cholerae* cells in the TB-treated loop was almost similar to that of the untreated loop (Fig. S4G).

**Fig 7:**
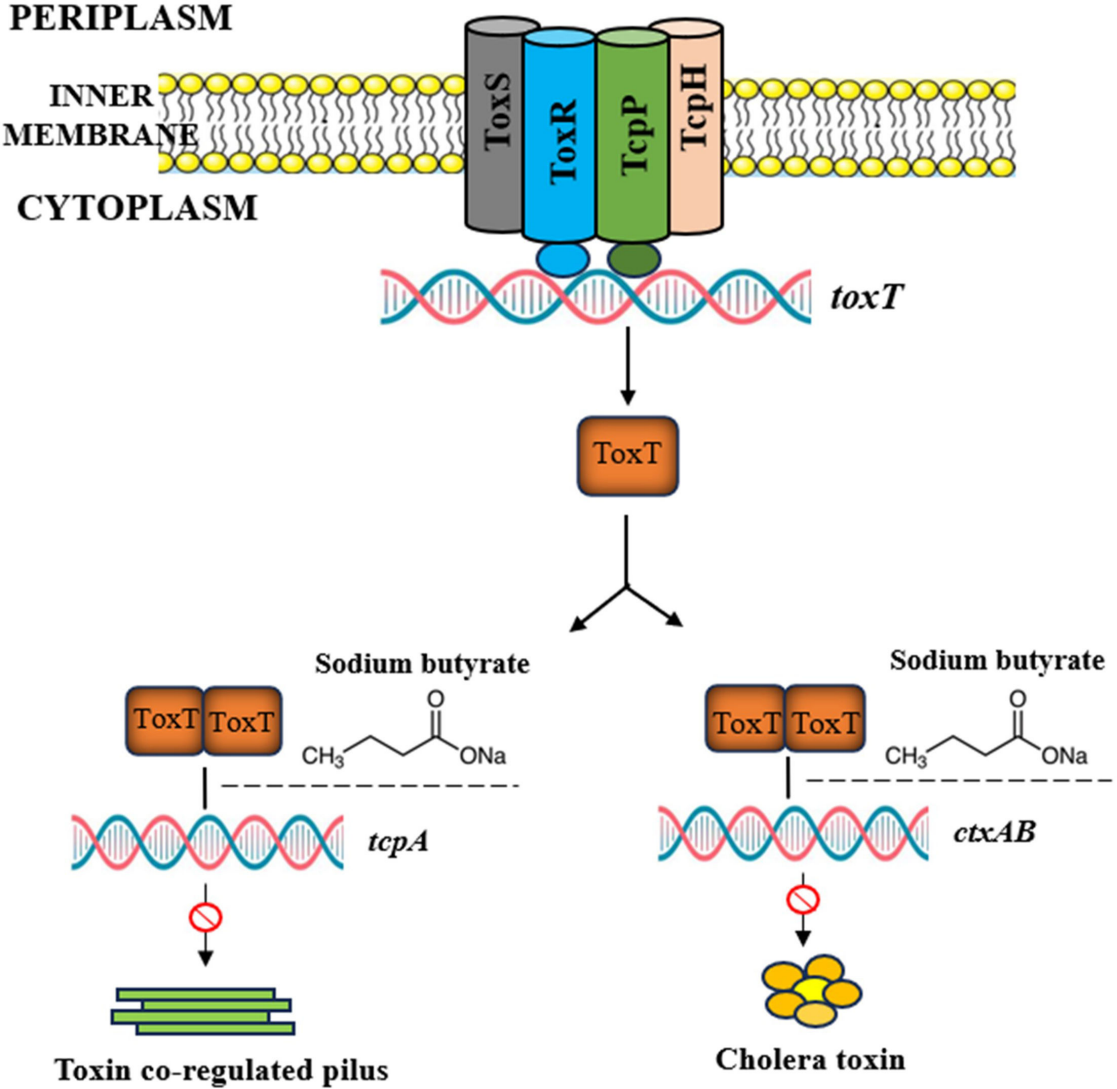
Model showing the inhibition of virulence cascade in *V. cholerae* by SB. The virulence cascade in *V. cholerae* is tightly regulated. TcpPH forms an inner membrane complex with ToxR and ToxS to activate the transcription of *toxT*. ToxT activates the transcription of *tcpA-F*, which encodes the toxin coregulated pilus, and *ctxAB*, which encodes the cholera toxin subunits. Based on our experiments, we propose that SB inhibits the binding of ToxT protein to the promoter region of *tcpA*/*ctxAB* and eventually inhibits cholera toxin and *tcpA* production.

The histopathological analysis of the infected ileal loop, stained with H&E, showed damage in the mucosa, submucosa, and lamina propria, along with disrupted villi and hemorrhage at the site of the muscularis mucosa. In contrast, the analysis of the ileal loops treated with SB displayed an almost normal microvilli structure. There were no significant alterations in the villi and mucosal structure, and no substantial damage was observed either in the submucosa or muscularis mucosa (Fig. 6E). Furthermore, loops that were subjected to SB treatment displayed a significant reduction in concentration of inflammatory cytokines (IL-6, IL-8, IL-1β, and TNF-α) compared with the loop that received no SB (Fig. S4C). In summary, SB downregulates the expression of *ctxAB* and *tcpA* genes by inhibiting ToxT activity (Fig. 7).

## DISCUSSION

Despite the use of antibiotics and oral rehydration therapy (ORT), *V. cholerae* remains a significant public health concern. This situation has emerged due to the increase of antibiotic-resistant *V. cholerae* strains, found in the majority of cholera cases, causing substantial mortality and morbidity (35). Developing new treatment therapies that target the key virulence factors without affecting the bacterial viability could be useful in managing these antibiotic-resistant strains (36). This study demonstrates that a small molecule, SB, effectively reduces the virulence potential of *V. cholerae in vitro* and *in vivo* conditions, without significantly affecting their viability.

In *V. cholerae*, the ToxR regulon plays a central role in the pathogenesis of the organism by regulating multiple virulence pathways (37). Of all the regulatory factors constituting the ToxR regulon, the ToxT protein directly activates the expression of major virulence factors *tcp* (toxin co-regulated pilus, expressing different adhesion proteins) and *ctx* (cholera toxin gene, expressing major toxin proteins like CtxA/B); therefore, it is considered a key target for the development of novel inhibitors (38). ToxT functions as a dimer with two well-defined functional domains in each monomer, a C-terminal DNA binding domain and an N-terminal regulatory/dimerization domain (39). Therefore, any compound that disrupts DNA binding and/or dimerization of ToxT would be expected to inhibit its activity. Based on our molecular docking analyses, SB was identified as a potential drug candidate that binds at the interface between the regulatory and DNA-binding domains of ToxT. SB was then experimentally tested to assess its impact on the ToxT-mediated transcription of *tcpA* and *ctxAB* genes. The results of our *in vitro* assays (EMSA and ChIP-qPCR) further confirmed that SB strongly reduced the expression of virulence genes by affecting the DNA binding ability of ToxT. Previous studies have shown a similar mechanism of action where unsaturated fatty acids (UFAs) and their conjugated forms exhibited anti-ToxT activity (22, 40).

Our RNA-seq data also provide evidence that SB attenuates the expression of *ctx* and *tcp* genes in a ToxT-mediated manner; however, an increase in the expression of biofilm genes was observed. This apparently conflicting result is not unprecedented. Previous studies have reported the ability of anti-virulence agents to downregulate the expression of *tcp* and *ctx* genes while simultaneously upregulating the expression of biofilm genes (41, 42). In addition, SB decreased the expression of accessory genes important for pathogenesis such as hemolysin (*hlyA*), accessory colonization factors (*acfA*), and enterotoxins (*zot*). Therefore, dysregulation of these virulence factors, particularly *ctx* and *tcp* genes, may reduce the pathogenicity of *V. cholerae.* Hence, targeting ToxT becomes even more significant in this context.

Prior studies have shown that when the anionic carboxylate of UFAs formed salt bridges with the two specific lysines (i.e*.*, Lys31 and Lys230) of ToxT, the protein was locked in a closed conformation and failed to dimerize and/or bind DNA (39, 43). Likewise, another study highlighted the importance of the carboxyl moiety of synthetic compounds, as it was found to be necessary for binding and inhibition of ToxT (44). In our present study, the docking analysis identified two key residues of ToxT, namely Lys31 (located at the N-terminal end) and Lys230 (situated at the C-terminal end) to be involved in SB-ToxT interaction. These two residues are predicted to form H-bonds and salt bridges with the anionic carboxylate group of SB. The absence of a free carboxyl group in TB may explain the molecule’s inability to interact with ToxT. Based on these observations, we speculate that interaction between the carboxyl group of SB and lysines of ToxT may play a crucial role in SB-ToxT stability, resulting in a direct blockage of protein-DNA binding and/or dimerization. Loss of activity triggered by ligand binding (SE-1, decanoic acid) is also well documented in other transcriptional factors such as VirF, Rns (45, 46).

There is a constant demand for alternative medicines that can be an efficient therapeutic to cure *V. cholerae* infections with minimal side effects on the host. A group of researchers identified a class of compounds, sulfonamides, which are widely used as bacteriostatic agents that inhibit cell growth by interfering with folic acid biosynthesis (47). Their spectrum of activity encompasses a wide range of gram-negative, gram-positive bacteria, and some protozoan species (47). In *V. cholerae*, sulfonamides exhibited anti-bacterial and anti-virulence activity by inhibiting metalloenzyme carbonic anhydrase, which reduced bicarbonate production, in turn affecting ToxT activity (major regulator of virulence factors in *V. cholerae*) (48–50). However, the main problem is that sulfonamides are promiscuous inhibitors of most human carbonic anhydrase isoforms, causing serious side effects like metabolic acidosis, kidney stones, and rare but severe conditions like Stevens-Johnson syndrome (SJS), toxic epidermal necrolysis (51). In the case of SB, several reports indicate that it exerts diverse beneficial effects on the host by maintaining intestinal homeostasis through changes in gene expression and signaling pathways (52, 53). It supports normal intestinal function by acting as an energy source for aerobic metabolism and increasing the proliferation of normal colonocytes (54). Butyrate also promotes blood flow and gut motility, which are important for digestion (53). Reports suggest that butyrate can alleviate obesity-related complications by significantly enhancing intestinal epithelial function, primarily by strengthening the gut barrier through tight junction regulation, thereby reducing inflammation and improving metabolic health (55, 56). Butyrate also possesses antibacterial activity against various gram-positive and gram-negative bacteria such as *Acinetobacter baumannii*, *Bacillus anthracis, Bacillus subtilis,* and *Staphylococcus epidermidis* (30). In our experiment, SB exhibited anti-virulence activity against *V. cholerae* by directly binding to ToxT and inhibiting its activity, which led to the downregulation of genes belonging to *tcp* and *ctxAB* operons. Although there are many benefits associated with SB for gut health, including its anti-bacterial and anti-virulence properties, it is important to acknowledge a few disadvantages associated with its use. First, studies have reported a paradoxical effect of butyrate on glucose and lipid metabolism, particularly in relation to its role in obesity. Although butyrate is reported to alleviate diet-induced obesity in mice (57), on the contrary, in a few studies, butyrate and other SCFAs have been found to contribute to the obese phenotype in humans by increasing lipid biosynthesis from acetyl CoA, FAs, and ketone bodies (58, 59). Second, butyrate may indirectly affect the host’s appetite and eating behavior by stimulating the vagus nerve and the hypothalamus, due to its capability to penetrate the blood-brain barrier (60, 61). Altogether, these reports suggest that SB is beneficial for gut health, but there are also a few side effects that may be manageable while developing targeted therapeutics to treat *V. cholerae* infections.

Butyric acid is a SCFA and one of the main metabolites of intestinal microbial fermentation of dietary fiber (62). Compared with other SCFAs, extensive research has been done on butyrate, which highlighted its importance in various pathological processes (63–65). As a U.S. Food and Drug Administration-approved drug (Butyric acid- 21CFR182.60), butyrate has been used in patients suffering from autoimmunity, cancer, and neurological diseases (66–68). Butyrate promotes intestinal epithelial barrier function and regulates the host mucosal immune system (69). Notably, butyrate is reported to limit pathogen proliferation through increasing mucosal barrier and secretion of AMPs (70). In our experiments, SB treatment decreased bacterial colonization in the suckling mice. Our findings are further supported by studies about the inhibitory effect of SB on the colonization of *Salmonella sp* (71, 72). Remarkably, SB treatment also reduced CT production and fluid accumulation in the rabbit ileal loop. However, when another butyrate derivative (TB) was used in animal models, it was ineffective against *V. cholerae* virulence. Since our *in vitro* findings demonstrated that SB inhibits ToxT activity while TB showed no such inhibition, we hypothesize that SB attenuates *V. cholerae* virulence *in vivo* possibly by targeting ToxT-mediated virulence. Altogether, these findings suggest that SB is highly effective against *V. cholerae* virulence, which may reduce the severity and duration of the disease. There are various reports regarding the *in vitro* inhibition of ToxT activity by the inhibitors, but their efficacy *in vivo* conditions remains far from being elucidated (40, 43). Here, we have shown for the first time that SB exhibited strong anti-virulence activity against *V. cholerae in vitro* and *in vivo* conditions.

Although we have demonstrated the protective effects of SB against *V. cholerae*, it is essential to acknowledge that our research currently possesses certain limitations and needs further investigation. First, in our study, a molecular docking experiment predicted the binding site of SB with ToxT. In addition to docking studies, a structural, in-depth analysis is requisite for gaining better insights into the exact binding position of SB and how this interaction affects the DNA binding ability. Second, our results so far do not offer a specific explanation for the increase in biofilm-forming genes. Future work will therefore aim to unravel the pathway(s) that can link the differential changes in virulence gene expression. Third, although we have explored the *in vivo* mechanisms of SB in suckling mice and rabbit ileal loops, these two animal models hold certain limitations. The lack of severe diarrhea and an underdeveloped host defense system in suckling mice do not provide information about the host factors important for the secretory response. In the case of the rabbit ileal loop, the closed intestinal loop system bypasses the natural route of infection as well as several aspects of GI tract physiology, such as peristalsis. Therefore, a direct confirmation of our study awaits an improved mammalian model.

In conclusion, this study reports that SB inhibits ToxT activity in *V. cholerae*. This is the first report of direct binding of SCFA, butyrate, to a virulence regulator in *V. cholerae*. Mitigation of *V. cholerae* virulence without reducing bacterial burden may also reduce the potential to develop unwanted antibiotic resistance. Furthermore, the homology of ToxT with other virulence regulators in different pathogenic bacteria could facilitate the development of broad-spectrum anti-virulent agents (46, 73). Such therapeutics would offer a new option to treat MDR bacterial infections.

## MATERIALS AND METHODS

### Computational methods

The 3D structure of the docking target ToxT was downloaded from the RCSB Protein Databank Server and visualized using UCSF Chimera version 1.11. The said protein was prepared for docking using the Yasara engine and then saved as a new PDB file using Chimera. The ligands used for docking were collected from different literature sources. Ligands were converted to the PDB file using UCSF Chimera (74, 75) and were energy minimized using universal force fields (UFF). These ligands were then docked into the binding pocket of ToxT using PyRx autodock vina, and the energy values were computed for each ligand. Ligands that fit into the binding pocket were selected and considered for further *in vitro* and *in vivo* studies.

### Bacterial strains and culture conditions

Bacterial strains used in this study and their antibiotic resistance profiles are listed in Table S2. Strains were maintained at −80°C in Luria-Bertani broth (LB) containing 20% glycerol. Overnight cultures were grown for 24 h at 37°C in LB medium. Growth of the *V. cholerae* strains under toxin-inducing conditions consists of diluting overnight culture 1:1,000 in fresh AKI medium, growing them under stationary conditions for an initial 4 h, and then shifting to shaking condition for another 16 h (15). Concentrations of antibiotics used (Sigma, Saint Louis, MO) were as follows: streptomycin, 100 µg/mL, and ampicillin, 100 µg/mL.

### Susceptibility testing and growth curve assay in the presence of SB

SB used in this study was purchased from Sigma-Aldrich. SB stock solution was made to 5 M in water and was stored at 4°C.

The MIC was determined following the Clinical and Laboratory Standards Institute microdilution assay using the cation-adjusted Mueller-Hinton broth (CAMHB) (76). Bacterial strains were grown on Luria agar plates (LA) at 37°C overnight. The colonies were resuspended in CAMHB to a 0.5 McFarland standard. The 100 µL of adjusted culture was further diluted with serially diluted concentrations of SB (ranging between 600 mM and 5 mM) in CAMHB in a round bottom 96-well plate to a final concentration of 5 × 10^5^ CFU/mL. The plates were incubated at 37°C for 24 h without shaking. The concentrations of SB that did not affect the bacterial growth were selected as the sub-MICs for this study, whereas the lowest concentration that inhibited the visible bacterial growth was identified as the MIC (77). To determine the MBC values, 100 µL of each well medium with no visible growth was inoculated in Mueller-Hinton agar plates (MHA). The lowest concentration at which no colonies were identified on the plate after 24 h incubation was determined to be the MBC.

To evaluate the effect of SB on bacterial growth, the overnight bacterial cultures were diluted 1:1,000 in a flask containing 200 mL of LB broth with or without SB and incubated at 37°C with shaking at 180 rpm. A 100 µL aliquot was removed from the flask, and suitable dilutions were plated on LA plates containing antibiotics. The growth curve was determined by cell counts and is expressed in log_10_CFU/mL. Experiments were independently performed three times.

### Detection of cholera toxin by ELISA

Cultures of *V. cholerae* were grown under toxin-inducing conditions in the presence or absence of SB. GM1 ganglioside enzyme-linked immunosorbent CT assays were performed as previously described (78) on equal volumes of resulting supernatant. CT expression values were normalized to OD_600_, and the average was calculated from triplicate experiments.

### RNA isolation and qRT PCR

Cells were cultured under toxin-inducing conditions in the presence or absence of SB. RNA was harvested with Trizol (Invitrogen, Carlsbad, CA) according to the manufacturer’s instructions, and DNA was removed using a DNA-free kit (Ambion, Austin, TX). RNA was converted to cDNA using the cDNA synthesis kit (Thermo Scientific, Waltham, MA). Real-time PCR was performed in the StepOnePlus real-time PCR system (Applied Biosystems, Foster City, CA) using SYBR green master mix (Applied Biosystems) according to the manufacturer’s instructions. The mRNA quantity relative fold change data were calculated using standard curves (79) and normalized by the expression levels of the *recA* gene (internal reference gene) (80). The results are the averages from three biological replicates with three technical replicates per experiment. Primer sequences used in this study are listed in Table S3.

### Western blot analysis

Cells were cultured under toxin-inducing conditions in the presence or absence of SB. Next, the bacterial cells were pelleted from each condition at 5,000  *× g* for 10  min. After the supernatant was decanted, the pellets were resuspended in phosphate-buffered saline (PBS). Cells were lysed by sonication for 3 min and centrifuged at 12,000  ×  *g* for 10  min at 4°C to remove cell debris. Then, the clear lysate was collected in a new tube, and protein concentrations were determined using a Bradford assay reagent (Thermo Scientific). Next, 20 µg of the total protein from each cell lysate was loaded and separated by 12% sodium dodecyl sulfate-polyacrylamide gel electrophoresis (SDS-PAGE). The proteins were transferred onto a polyvinylidene fluoride (PVDF) membrane (Millipore, Burlington, MA) and probed via immunoblotting. The following antibodies were used for immunoblotting: rabbit polyclonal ToxT, TcpA, and DnaK (Biobharati Life Sciences, West Bengal, IN) primary antibodies and horseradish peroxidase (HRP)-conjugated goat anti-rabbit secondary antibody (Sigma-Aldrich). Bands were observed in the ChemiDoc MP Imaging System (Bio-Rad, Hercules, CA) using chemiluminescent HRP substrate (Millipore). Relative fold changes in protein expression were measured after normalizing against DnaK using Image Lab software (version 5.2.1).

### Protein purification

Nickel binding protein-recombinant protein fusion (NBP-ToxT/CytR) purification was performed as previously described using *Escherichia coli* strain BL21(DE3) with plasmid pHIS-Tev plasmid harboring the 6× His-recombinant protein fusion construct (40). Briefly, after NBP-recombinant protein fusion construct induction by Isopropyl-β-d-thiogalactopyranoside (IPTG), cells were lysed by sonication and loaded on top of Ni^2+^-conjugated agarose beads. The fractions containing NBP-recombinant protein were dialyzed with 25 mM Tris (pH 8.0) and 100 mM NaCl solution. Purified protein was quantified by Bradford reagent, and the purity was confirmed by SDS-PAGE, stained with Coomassie Brilliant Blue.

### Fluorescence quenching

Interaction between ToxT protein and SB was analyzed by the concentration-dependent effect of SB on the tryptophan fluorescence emission of ToxT. The emission spectra of ToxT were acquired using 0.1 µM protein dissolved in 25 mM Tris (pH 7.5) and 100 mM NaCl in the presence or absence of SB. The solution was excited at 280  nm, and the intrinsic fluorescence emissions were scanned from 300 nm to 500 nm (Hitachi Fluorescence Spectrophotometer F-7000, Tokyo, Japan). Fluorescence quenching was analyzed by the classical Stern-Volmer equation as previously described (81).

### EMSA and ChIP

The chromosomal DNA of *V. cholerae* N16961 was used as a template in the PCR process to amplify DNA fragments of size 150 bp for the *tcpA* promoter region. Then this amplified product was labeled with biotin at the 5′ end followed by purification using the Qiaquick nucleotide removal kit (QIAGEN, Hilden, Germany). EMSA of ToxT protein binding to the *tcpA* promoter region was performed as described previously (22). For the EMSA binding assay, different concentrations of purified ToxT protein (0.4, 0.6, 0.8, 1.0, 1.2, 1.4, and 1.6 µM) were mixed with fixed amounts of biotin-labeled DNA fragment *P_tcpA_* (1 nM) in a binding buffer (10 mM Tris-HCl [pH 7.5], 100 mM KCl, 1 mM EDTA, 1 mM dithiothreitol, 200 µg of bovine serum albumin/mL, and 10% glycerol) and incubated for 20 min (82). To check the SB-ToxT interaction, the binding reactions were set up where different concentrations of ToxT (0.4, 0.6, 0.8, 1.0, 1.2, 1.4, and 1.6 µM) were pre-incubated with SB (20 mM) for 20 min in binding buffer, followed by the addition of DNA fragment *P_tcpA_* (1 nM) and incubation continued for another 20 min. To determine the effect of increasing concentrations of SB, the binding reactions were set up where ToxT (1.0 µM) was pre-incubated with varying doses of SB (10, 20, and 40 mM) for 20 min in binding buffer, followed by the addition of DNA fragment *P_tcpA_* (1 nM). To determine the specificity of SB-ToxT interaction, the binding reactions were set up where ToxT (1.0 µM) was pre-incubated with SB (40 mM) or another butyrate derivative TB (40 mM) for 20 min in binding buffer, followed by the addition of DNA fragment *P_tcpA_* (1 nM). Furthermore, to check possible SB-*P_tcpA_* DNA interaction, labeled *P_tcpA_* DNA (0.5 nM) was pre-incubated with SB (20 and 40 mM) for 20 min in binding buffer, followed by the addition of purified ToxT protein (4 µM), and the incubation continued for another 20 min (82). To show the specificity, non-specific protein (CytR, 1.0 µM), a 70-fold molar excess of unlabeled double-stranded *tcpA* fragment, and a 70-fold molar excess of unlabeled nonspecific DNA were used as controls (Table S3). The samples were loaded on 4% native polyacrylamide gel and electrophoresed at 100 V, 4°C in 0.5× Tris-borate-EDTA buffer. DNA was then transferred from gel to charged nylon membrane for nucleic acid blotting (Millipore) followed by cross-linking using a UV Stratalinker from Stratagene and detected using LightShift Chemiluminescent EMSA Kit (Thermo Scientific).

To determine the *K_d_* (equilibrium dissociation constant) for samples with or without SB, the percentage of labeled DNA bound to protein was determined for each lane. This was then fit to the following equation: percent bound = *B*_max_ × [protein]*^h^*/(*K_d_^h^* + [protein]*^h^*), where *h* is the Hill coefficient and *B*_max_ is the amount of bound DNA at which the curve plateaus, which was set to a constraint of 100% using GraphPad Prism 9.0 software. The *K_d_* values for each condition were compared using the extra sum of squares F test to determine if the two values were statistically different.

ChIP analysis was performed as described previously (83), with some modifications. Briefly, cells were grown in the presence or absence of SB for 4 h. After cross-linking with formaldehyde, cells were lysed and sonicated to shear the genomic DNA. Clarified lysates were incubated for 6 h at 4°C with 8 µg anti-ToxT antibody or control mouse IgG (Cell Signaling Technology). The chromatin fraction, which lacks the primary antibody, was taken as “input’.’ After washing, the immunoprecipitated complexes were eluted, and DNA was reverse cross-linked. Real-time quantitative PCR (qPCR) and agarose PCR gel electrophoresis were used to quantitate promoter occupancy by ToxT as formerly described (84, 85) using the primers for ChIP (Table S3). The data were graphically represented as % input.

### RNA sequencing and analysis

Triplicate N16961 samples were grown under toxin-inducing conditions in the presence or absence of SB. Total RNA was isolated as described above, and mRNA was selectively enriched by depleting rRNA using a Ribo-Zero rRNA removal kit (Epicentre, Madison, WI). Then, the cDNA library was constructed using a TruSeq-stranded mRNA sample kit (Illumina, San Diego, CA). RNA sequencing was done on the Illumina HiSeqX system by MedGenome Labs Ltd., Bangalore, India. The raw data were processed using the HISAT2-StringTie pipeline as described elsewhere (86). The raw sequencing reads were mapped against the genomic sequence of *V. cholerae* O1 biovar El Tor str. N16961 using the HISAT2 alignment tool. StringTie was used to assemble reads and generate FPKM (fragments per kilobase per million) values, as a normalization metric (87). After filtering undesirable contaminants (such as rRNAs), differentially expressed genes between different groups were identified by the package DESeq2 [20 mM of SB over 0 mM of SB treatment were determined as fold change (FC) by the formula log2(mean FPKM_test_/ mean FPKM_control_)], with thresholds of adjusted *P* values at ≤0.05 and an absolute fold change ≥2. The differentially expressed genes identified among the two groups were generated in a heatmap by the CIMminer program as described previously (88). The complete list of the FPKM values, fold change values, and *P* values are provided in Data Set S1.

### Cell culture experiments

Human adenocarcinoma cell line HT-29 (ATCC HTB-38) was maintained in Dulbecco modified essential medium (DMEM) (Sigma-Aldrich) supplemented with 10% fetal bovine serum (PAN Biotech, Aidenbach, BY). The cells were kept at 37°C in a humidified 5% CO_2_ incubator.

Adhesion of *V. cholerae* to HT-29 cells was done according to the previously described method (80). Briefly, HT-29 cells were grown up to 80%–90% confluency. The cells were infected with bacterial suspensions in DMEM (without antibiotics) at an MOI of 100 for 2 h in the presence or absence of SB. Following incubation, the nonadherent bacteria were completely removed by washing with PBS. Cells were lysed in 0.1% Triton X-100 solution. The lysate solution was gradient-diluted and plated on LA plates, and the plates were incubated overnight at 37°C. The attachment efficiency was determined by the number of bacteria recovered on plates. At least three independent biological replicates were prepared and analyzed.

The MTT assay was performed as described previously (89). Briefly, HT-29 cells were treated with different doses of SB (5–160 mM) at 37°C. The cell viability was determined after 24 h using the Colorimetric Cell Viability Kit IV (MTT) (Promokine, Heidelberg, Germany) according to the manufacturer’s guidelines. The absorbance was read at 570 nm, and % viability was calculated as described previously (26).

### Suckling mice colonization assay

Four- to five-day-old suckling BALB/c mice were orogastrically inoculated with 50 µL (containing 10^5^  CFU of *V. cholerae* cells) of the bacterial suspension with or without drugs. The mice were maintained at 30°C and sacrificed after 18  h. The entire intestine was removed and homogenized in PBS. Serial dilutions of the homogenates were then plated on LA plates containing antibiotics to enumerate viable *V. cholerae* cells and expressed as CFU per mouse intestine (90). The colonization experiment was performed three times independently in 3 days with at least four mice in each group, and the combined data for the three experiments were used for statistical analysis.

### Rabbit ileal loop assays

Assays of fluid accumulation in rabbit ileal loops were performed as previously described (90). Each loop received 1 mL (containing 10^9^  CFU of *V. cholerae* cells) of bacterial suspension with or without drugs. After 18 h, the rabbits were sacrificed, and their intestine was dissected. The fluid accumulated within each loop was collected separately, measured, and expressed as a ratio of the amount (mL) of fluid per unit length (cm) of the loop. The amount of CT produced was assessed by CT-ELISA, and the inflammatory cytokines (IL-6, IL-8, IL-1β, and TNF-α) produced under each condition were determined by ELISA kits (Krishgen, Mumbai, India) following the manufacturer’s instructions. For qRT-PCR analysis of virulence genes, bacteria were harvested from loop fluid by centrifugation and used for RNA preparation, which is further processed to qRT-PCR as described above.

The ileal loops obtained from the above procedure were analyzed for the estimation of adhered bacteria to the intestinal mucosa following the previously described protocol (91). Briefly, the fluid in the loops was taken out as outlined above. Each intestine was then excised and opened by longitudinal incision and was washed three times with PBS to remove the nonadherent or loosely adhered bacteria. After washing, the opened intestine was stretched on a wooden sheet with a luminal surface uppermost, and several circular pieces of mucosa (7 mm in diameter) were punched out. Each of the intestinal punches was then homogenized in 0.25 mL of Krebs/Ringer/Tris (KRT) buffer (pH 7.5). To determine the viable cells of *V. cholerae*, 0.1 mL of the homogenate was serially diluted in PBS and 0.1 mL of aliquots were plated onto LA plates containing antibiotics to enumerate viable *V. cholerae* cells. The adhesive ability of *V. cholerae* (adherence index) was expressed as the average number of adhered bacterial cells per punched mucosal surface.

For the histopathological study, rabbit ileal tissue samples were fixed with 10% buffered formalin, embedded in paraffin, and sliced into 4-μm-thick sections. The tissue was then deparaffinized and stained using HE and visualized using a bright field microscope for further analysis. The rabbit ileal loop experiments were performed three times independently, and the results were expressed as mean ± SD (*n* = 3).

### Statistical analysis

Statistical analyses were carried out using the GraphPad Prism 9.0 software, and all data are denoted as mean ± standard deviation (SD) unless otherwise specified. The results were analyzed using appropriate statistical tests as indicated in figure legends.

## Data Availability

All raw transcriptome data have been deposited in the NCBI BioProject database under accession number PRJNA1194555.

## SUPPLEMENTAL MATERIAL

DATA SET S1 msphere.00824-24-s0001.xlsx RNA seq.

FIGURE S1 msphere.00824-24-s0002.tif Susceptibility testing and cytotoxicity of SB

FIGURE S2 msphere.00824-24-s0003.tif Effect of SB on the virulence attributes of V. cholerae.

FIGURE S3 msphere.00824-24-s0004.tif EMSAs demonstrating the specificity of the interactions.

FIGURE S4 msphere.00824-24-s0005.tif Efficacy of drugs against the virulence factors and inflammatory cytokines *in vivo*.

Supplemental material msphere.00824-24-s0006.docx Supplemental figure legends and tables.

ASM does not own the copyrights to Supplemental Material that may be linked to, or accessed through, an article. The authors have granted ASM a non-exclusive, world-wide license to publish the Supplemental Material files. Please contact the corresponding author directly for reuse.
