# Supplementary figures and images for: Sodium butyrate inhibits the expression of virulence factors in Vibrio cholerae by targeting ToxT protein
Source: mSphere. 2025 Apr 22;10(5):e00824-24. doi: 10.1128/msphere.00824-24 (PMC12108080; doi:10.1128/msphere.00824-24)

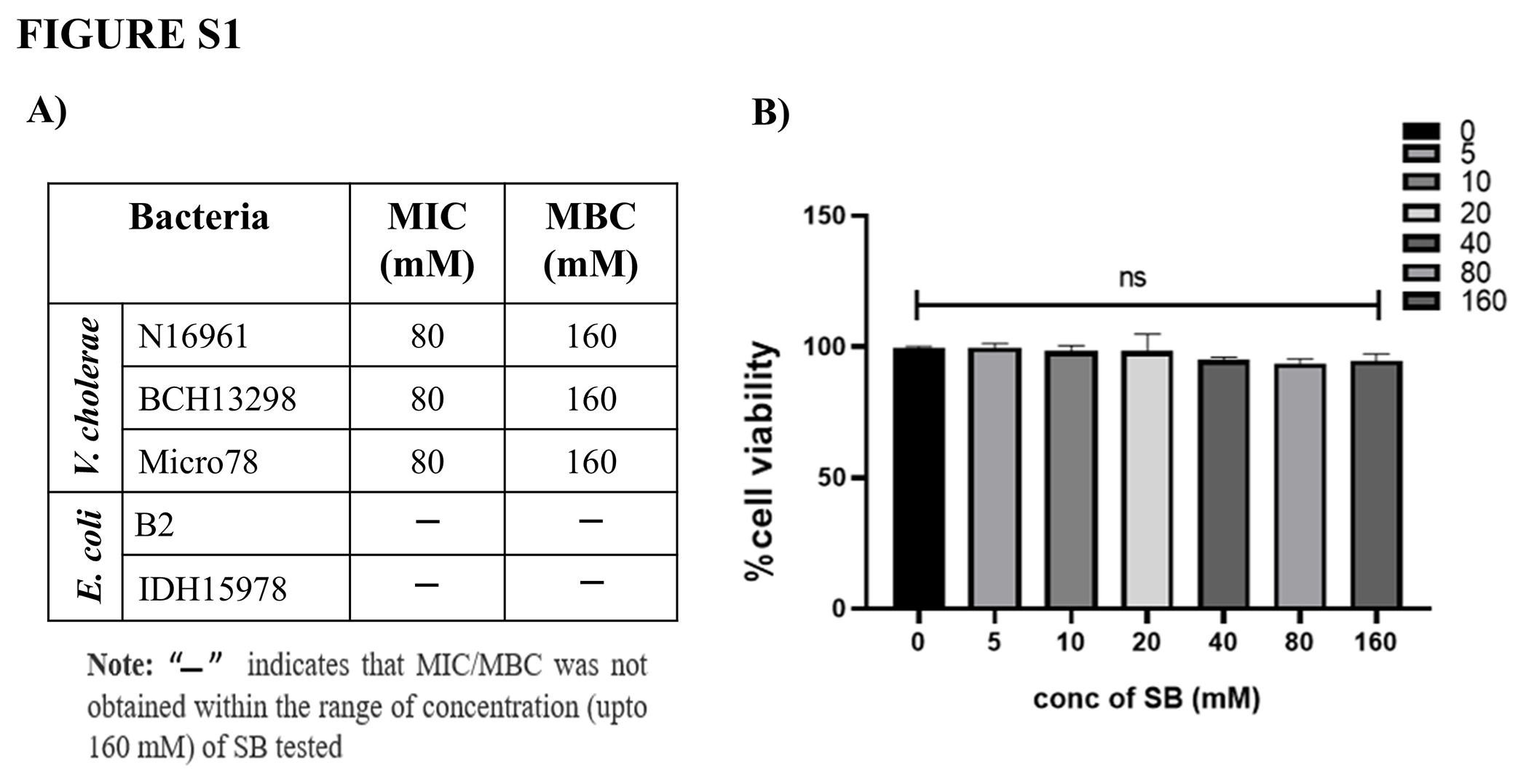

Supplement: FIGURE S1 — Susceptibility testing and cytotoxicity of SB [file msphere.00824-24-s0002.tif]

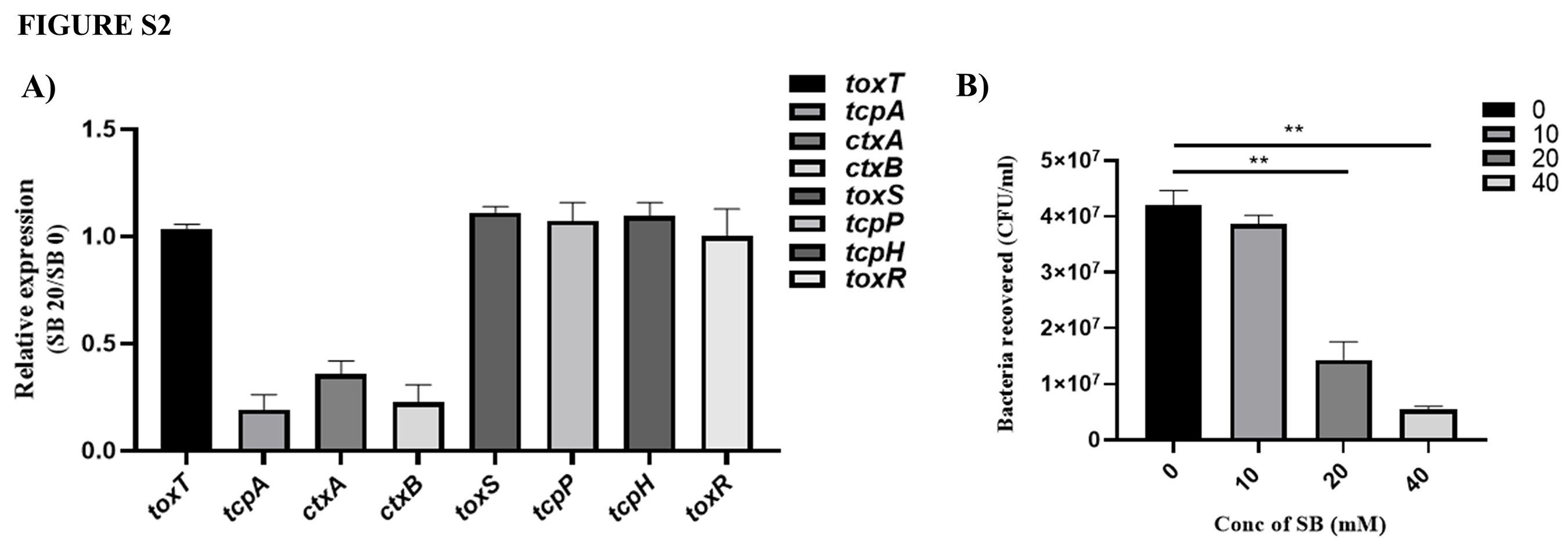

Supplement: FIGURE S2 — Effect of SB on the virulence attributes of V. cholerae. [file msphere.00824-24-s0003.tif]

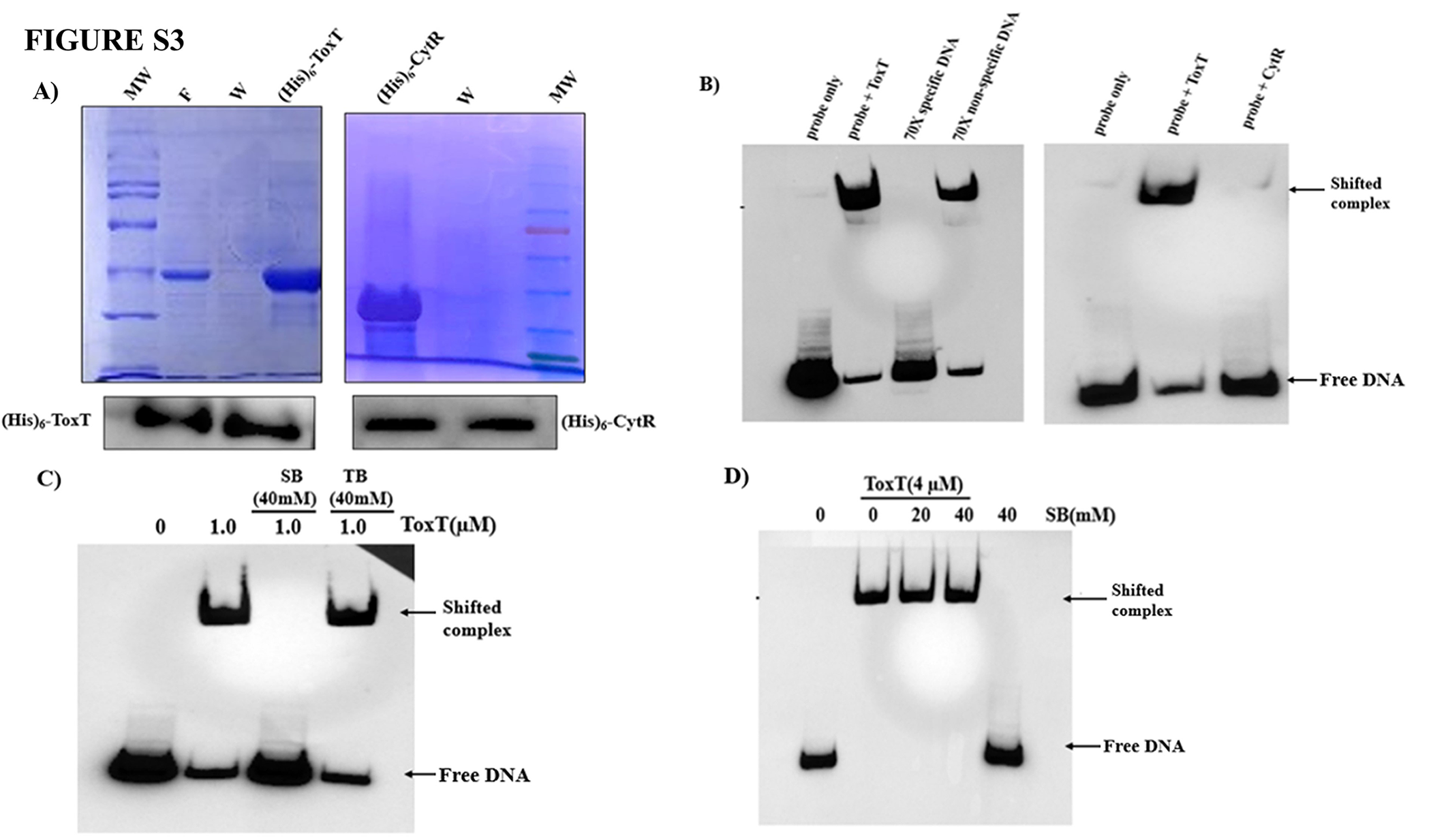

Supplement: FIGURE S3 — EMSAs demonstrating the specificity of the interactions. [file msphere.00824-24-s0004.tif]

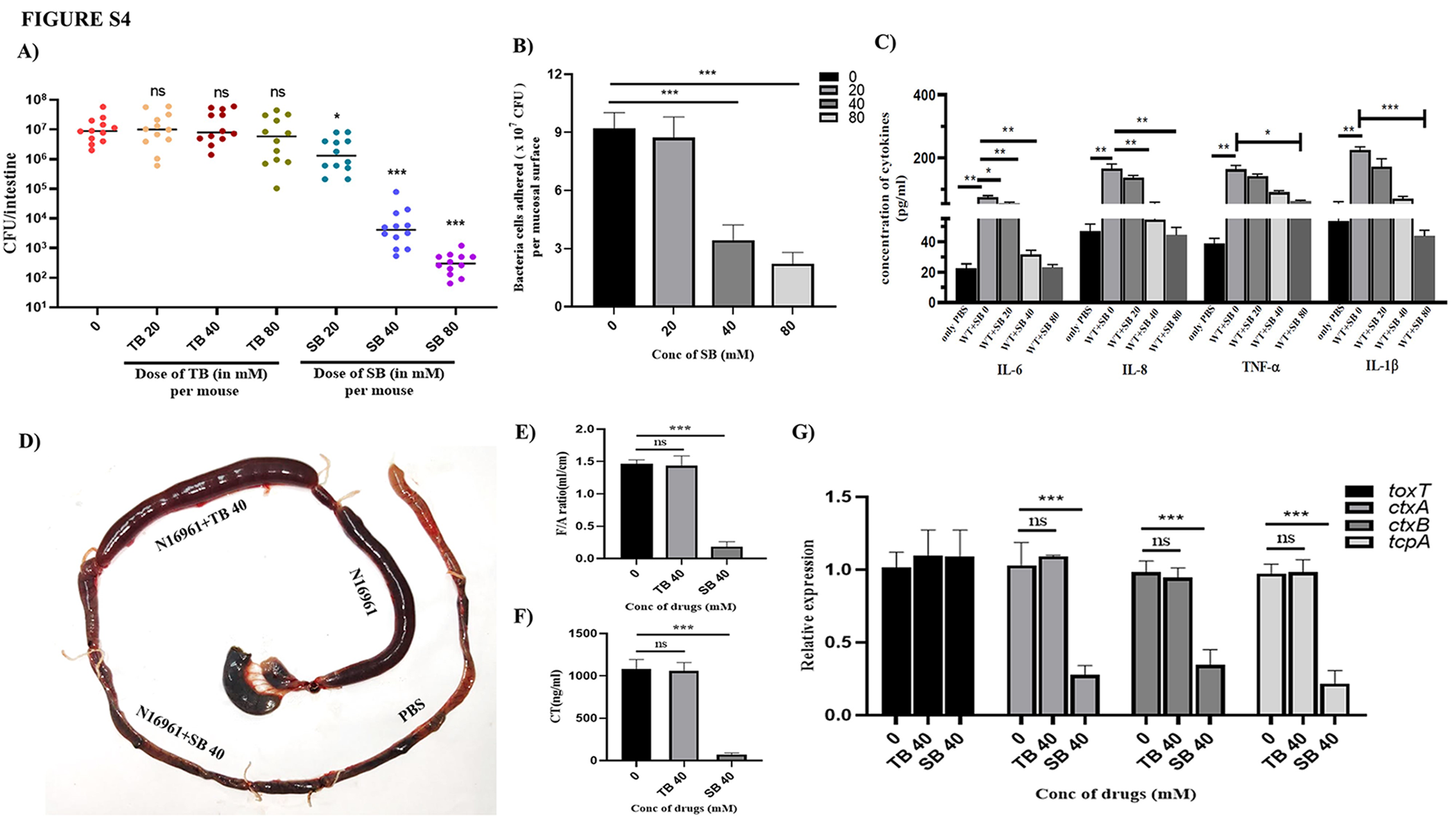

Supplement: FIGURE S4 — Efficacy of drugs against the virulence factors and inflammatory cytokines in vivo. [file msphere.00824-24-s0005.tif]
