# Supplementary material for: Sodium butyrate inhibits the expression of virulence factors in Vibrio cholerae by targeting ToxT protein
Source: mSphere. 2025 Apr 22;10(5):e00824-24. doi: 10.1128/msphere.00824-24 (PMC12108080; doi:10.1128/msphere.00824-24)
Supplement: Supplemental material — Supplemental figure legends and tables. [file msphere.00824-24-s0006.docx]

**FIGURE LEGENDS**

**Fig.S1) Susceptibility testing and cytotoxicity of SB. A)** Determination of MIC and MBC of SB against bacterial strains. **B)** The HT-29 cells were treated with or without SB (5-160mM) for 24 h. MTT assay was performed to measure the % viability level. One-way ANOVA was performed, and the significance was calculated as ns=nonsignificant. FIGURE-S1.tif

**Fig.S2) Effect of SB on the virulence attributes of *V. cholerae*. A)** Effects of SB on the expression of virulence genes (*ctxA*(**_▀_**), *ctxB*(**_▀_**), *toxS*(_▀_)*, toxR*(_▀_)*, tcpH*(_▀_)*, tcpP*(_▀_)*, tcpA*(**_▀_**)*, toxT*(**_▀_**)) in *V. cholerae*. *V. cholerae* N16961 cells were treated with or without SB, and virulence gene expression was determined by real-time quantitative PCR using specific primer pairs (Table S3). The values represent the number of times the genes are expressed compared to the untreated cells. **B)** Adherence of *V. cholerae* to HT-29 cell line performed at different concentrations of SB (0-40 mM). One-way ANOVA was performed. Significance levels were denoted as ∗∗for *P <* 0.01. FIGURE-S2.tif

**Fig.S3) EMSAs demonstrating the specificity of the interactions. A)** Protein purification and western blot analysis. Coomassie blue staining of the purification of (His)_6_-ToxT/(His)_6_-CytR fusion protein using nickel chelated affinity chromatography. Mw, molecular weight marker; F, flow-through fraction; W, washed fraction. Western blot of ToxT/CytR protein using anti-His tag antibody. **B)** ToxT binds specifically to P*_tcpA_*_._ ToxT (1.0 µM) was incubated with 1nM of biotin-labeled P*_tcpA_* in the reaction buffer in the presence or absence of either 70x specific or non-specific competitors. 1nM of biotin-labeled P*_tcpA_* was incubated with ToxT (1.0 µM) or CytR (1.0 µM) in the reaction buffer. **C)** ToxT interacts with SB but not with TB. ToxT (1.0 µM) was incubated with 40mM Sodium Butyrate (SB) or 40mM Tributyrate (TB) followed by the addition of P***_tcpA_*** (1nM). **D)** SB binds with ToxT but not with DNA. Biotin labeled P***_tcpA_*** DNA (0.5nM) was incubated with SB (20 and 40mM) followed by ToxT protein (4 µM) addition. The EMSAs presented are representative of three independent experiments. FIGURE-S3.tif

**Fig.S4)** **Efficacy of drugs against the virulence factors and inflammatory cytokines *in vivo****.* **A)** Effect of drugs on bacterial colonization in 4 to 5-day-old suckling BALB/c mice (*n*=12 per group). The mice were orogastrically challenged with 10^5^ CFU of *V*. *cholerae* strain N16961 with or without drugs [TB or SB (20, 40, and 80 mM)] and kept at 30ºC for 18 hours. Bacterial colonization was estimated as CFU/intestine and graphically represented. Each circle represents an individual mouse. Horizontal lines are medians. Significance was determined by Mann–Whitney *U* test (*n*=12 per group). Significance levels (compared to controls challenged without any drug) were denoted as ns=nonsignificant, ∗ for *P <* 0.05, ∗∗∗ for *P <* 0.001. **B)** The rabbit ileal loop was injected with 10^9^ CFU per ml *V. cholerae* N16961 with or without SB. After 18 hrs, the animals were euthanized, the loops were removed and adherence index of N16961 were estimated in the presence or absence of SB. Adherence index is denoted by the average number of adhered bacterial cells per punched mucosal surface where each punch was 38.5 mm^2^ (7 mm diameter). **C)** ELISA results for IL-6, IL-8, IL-1β, and TNF-α cytokine expression in each of the ileal loops in the presence or absence of SB. **D)** Each loop was injected with 10^9^ CFU per ml *V. cholerae* N16961 with or without drugs [TB or SB (40 mM)]. After 18 hrs, the animals were euthanized, and the loops were removed. The image of the recovered rabbit intestine segment presented here is representative of three independent experiments. **E)** The loop length and the amount of fluid accumulated in each loop were measured, and the amount of fluid (ml) per unit length (cm) of the loop was determined in the presence or absence of drugs [TB or SB (40 mM)]. **F)** CT ELISA of rabbit ileal loop fluid produced in the presence and absence of drugs [TB or SB (40 mM)]. **G)** Relative expression of major virulence genes *ctxA* (■), *ctxB* (■), *tcpA* (■), *toxT* (■) were analyzed by real-time PCR. Significance was calculated by one-way ANOVA. Significance levels were denoted as ∗for *P <* 0.05, ∗∗for *P <* 0.01, ∗∗∗ for *P <* 0.001. FIGURE-S4.tif

**TABLE S1: List of bioactive compounds that were screened against ToxT protein.**

| **Bioactive compounds** | **Uses** | **Structure** | **Binding energy (Kcal/mol)** |
| --- | --- | --- | --- |
| 3-chlorocatechol | Anti-virulence | 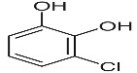 | -0.9 |
| phenoxyacetamide | Anti-viral | 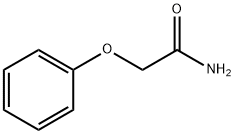 | -0.1 |
| sorafenib | Antineoplastic | 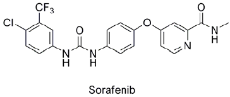 | -0.2 |
| Tributyrate | Anti-bacterial | 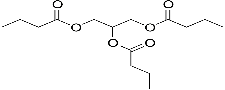 | -1.8 |
| Melibiose | Anti-bacterial | 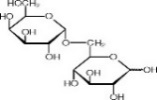 | -1.4 |
| Caffeine | Anti-virulence | 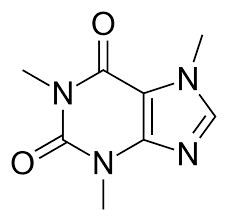 | -0.3 |
| Ribose | Anti-virulence | 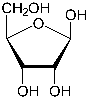 | -1.3 |
| Colibactin | Antineoplastic | 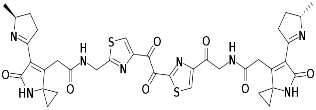 | -0.8 |
| axitinib | Antineoplastic | 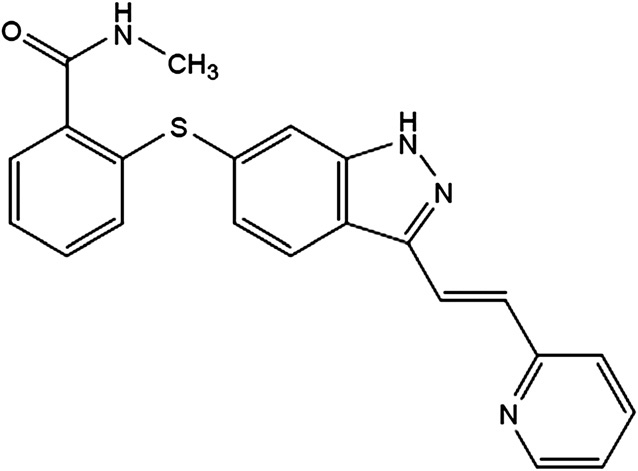  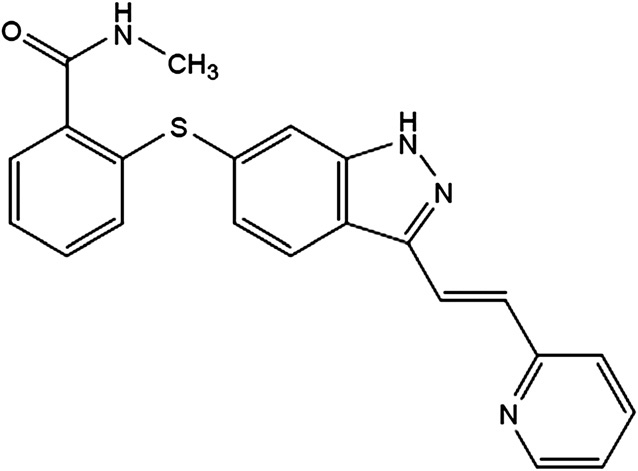 | -0.9 |
| hydroxyemodin | Anti-virulence | 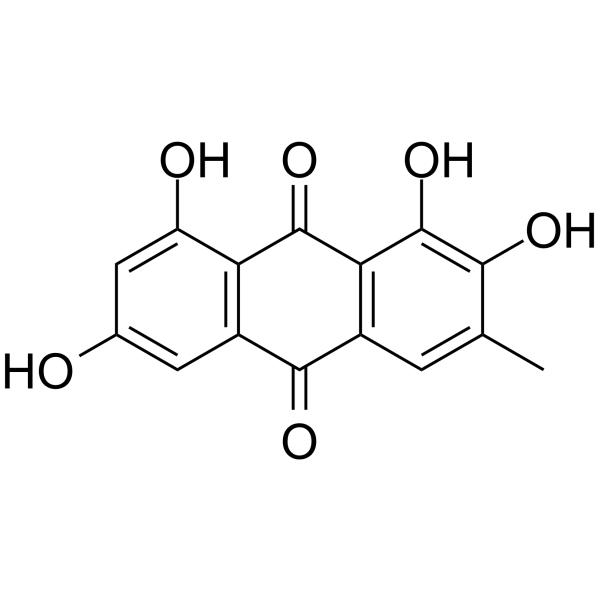 | -1.3 |
| phloretic | Antifungal | 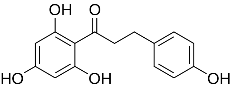 | -1.2 |
| geraniol | Antioxidant | 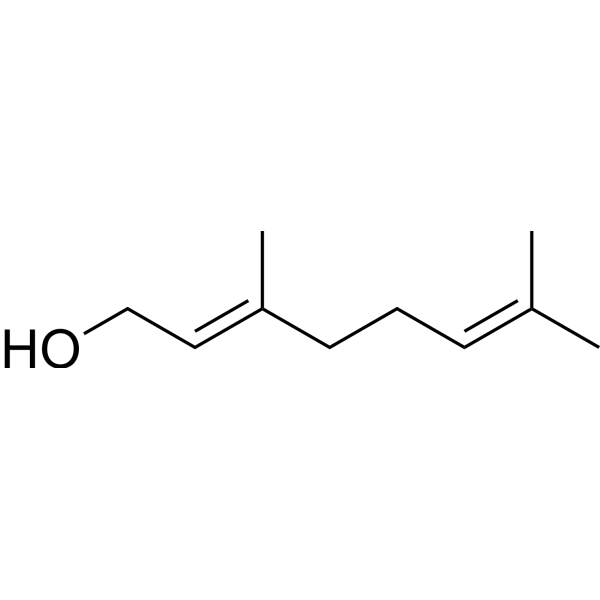 | -0.8 |
| NH125 | Antibacterial | 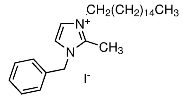 | -1.2 |
| pentoxifylline | Anti-virulence | 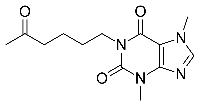 | -0.2 |
| Dequalinium chloride | Antimicrobial | 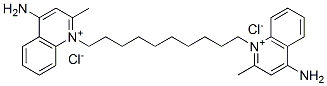 | -1.5 |
| Theophylline | Antibacterial | 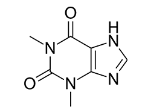 | +1.1 |
| Ethyl gallate | Antioxidant | 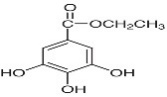 | -0.6 |
| glucose | Antibacterial | 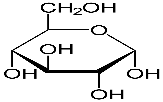 | +2.5 |
| **Bioactive compounds** | **Uses** | **Structure** | **Binding energy (Kcal/mol)** |
| GlcNAc | Anti-inflammatory | 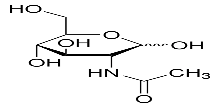 | -1.6 |
| Sodium butyrate | Anti-inflammatory | 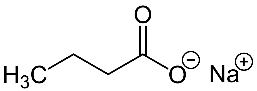 | -7.68 |
| mannose | Anti-virulence | 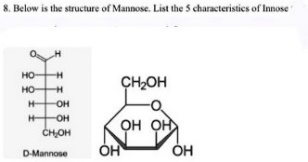 | -0.1 |
| 7-fluoroindole | Anti-virulence | 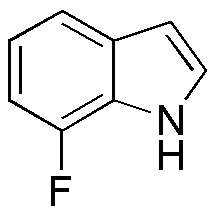 | +5.9 |
| quercetin | Anti-inflammatory | 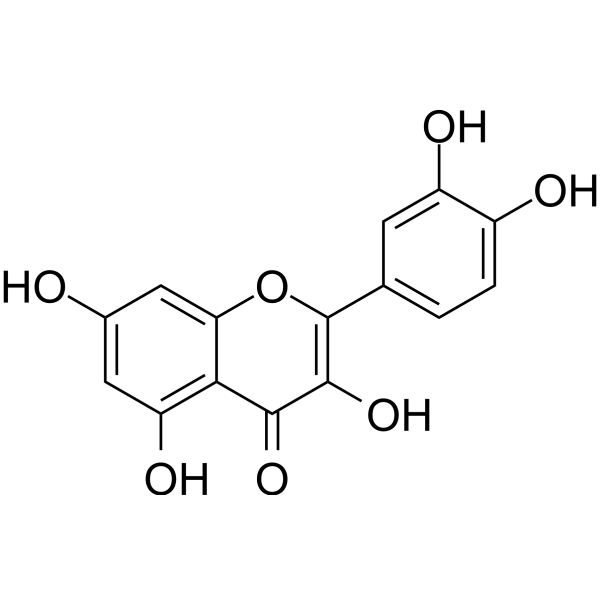 | -1.5 |
| ebselen | Anti-inflammatory | 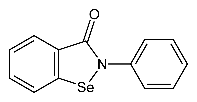 | -0.8 |
| cellobiose | antioxidant | 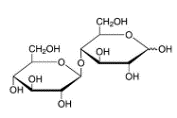 | -1.6 |
| xylitol | Antidiabetic | 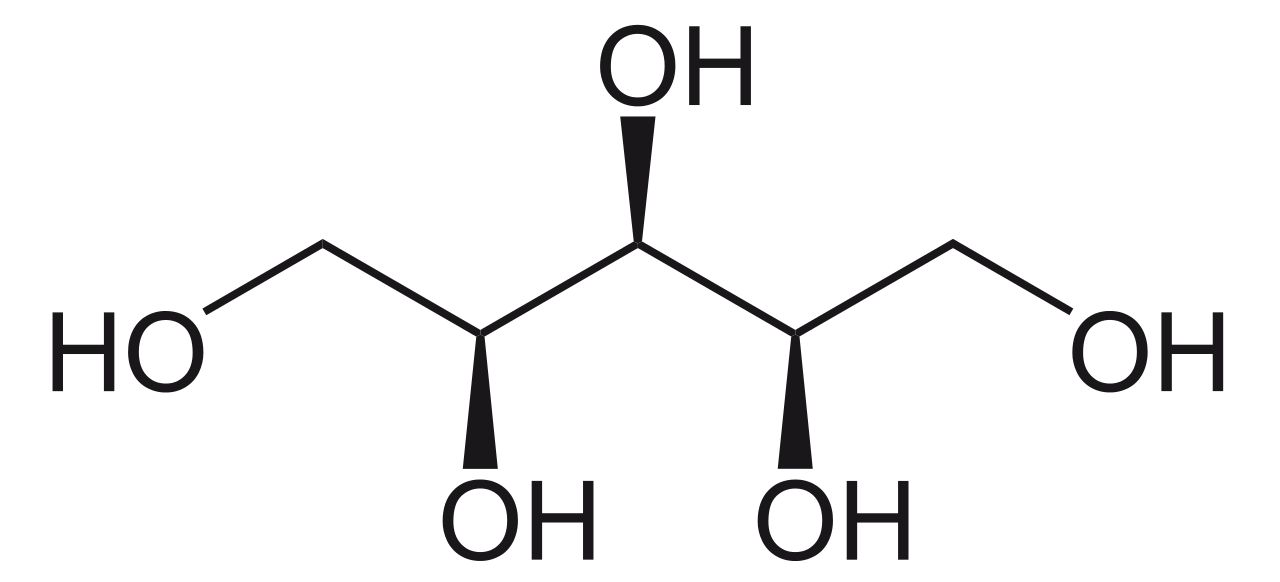 | -0.9 |
| Dantrolene sodium | Antispasmodic | 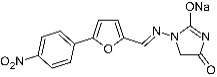 | -0.4 |
| Chalcone | Antidiabetic | 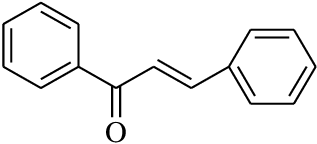 | -0.6 |
| Theobromine | antimicrobial | 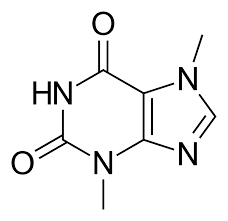 | -0.8 |
| Dapsone | antibacterial | 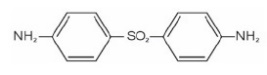 | -1.2 |
| Disulfiram | antimicrobial | 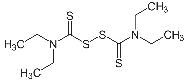 | -0.3 |
| Octodrine | antifungal | 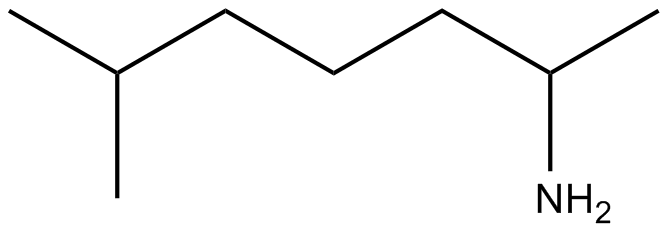 | -1.6 |
| Niclosamide | Anti-helmintic | 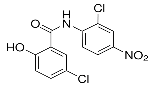 | -0.3 |
| Closantel | antiparasitic | 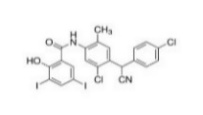 | +2.1 |
| Carmofur | antineoplastic | 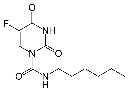 | -0.4 |
| Floxuridine | antineoplastic | 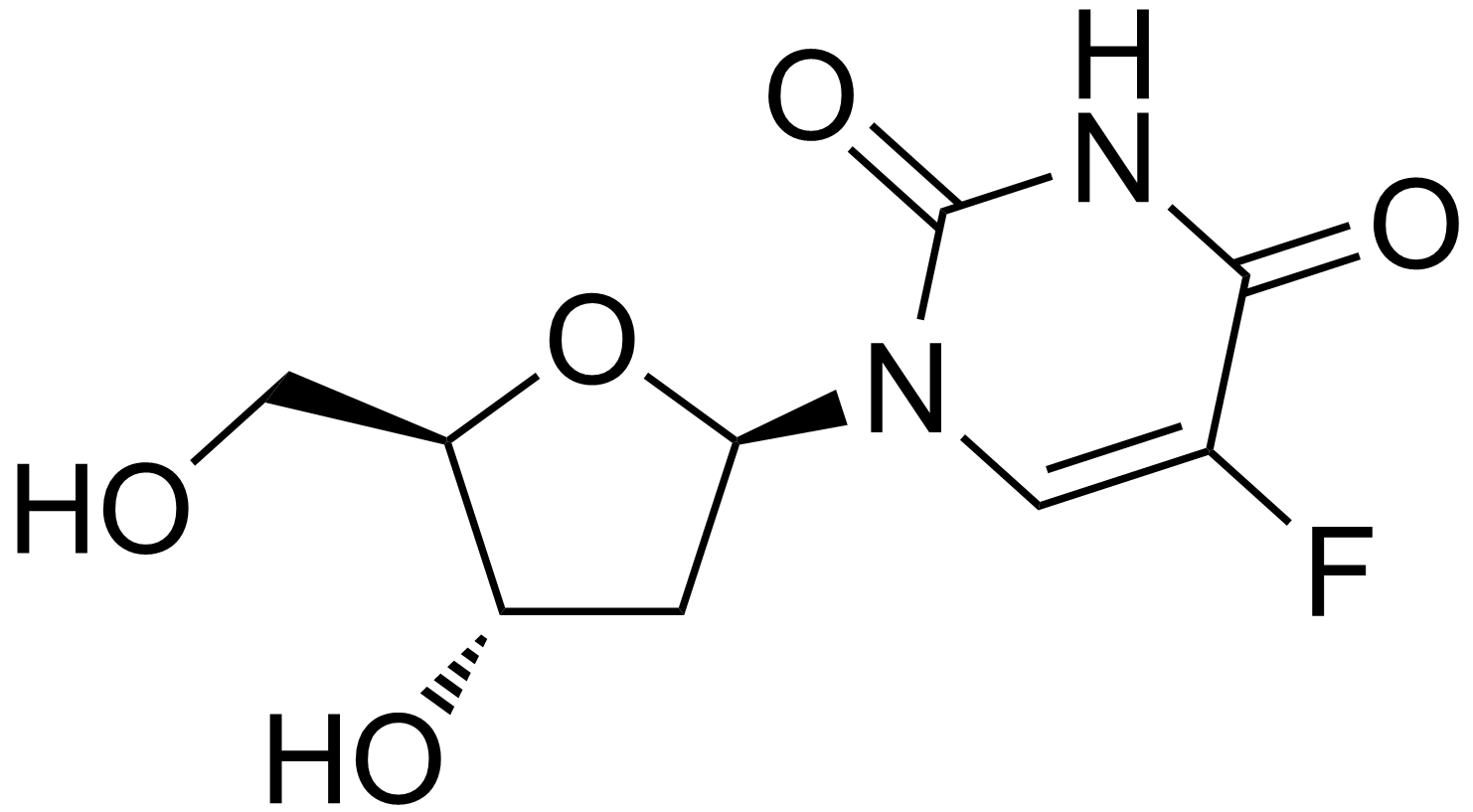 | +5.8 |
| gemcitabine | antineoplastic | 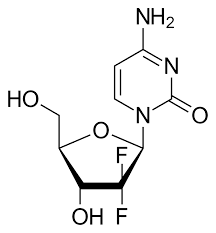 | -1.5 |
| Benzbromarone | Anti-virulence | 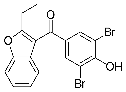 | -1.7 |
| anthrone | Anti-bacterial | 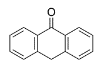 | -0.4 |
| Tolcapone | Antiparkisonian | 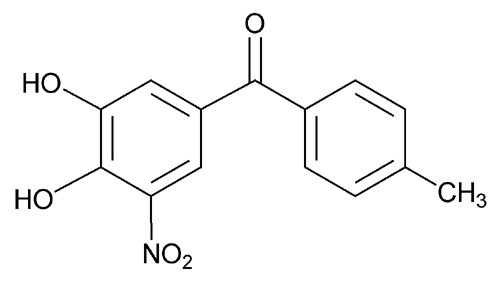 | -0.6 |
| **Bioactive compounds** | **Uses** | **Structure** | **Binding energy (Kcal/mol)** |
| butenolide | Anti-virulence | 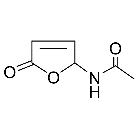 | -0.8 |
| oroidin | antibacterial | 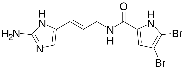 | -0.7 |
| lamotrigine | antibacterial | 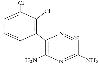 | -0.9 |
| p-allylanisole | antibacterial | 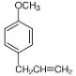 | +1.2 |

**TABLE S2: Antibiogram profile of bacterial strains determined according to the CLSI guidelines** (1)**.**

| **Name of the antibiotics (Disc content µg/disk)** | **Zone diameter interpretative criteria (CLSI guidelines) mm** | | ***V. cholerae* strains (zone size in mm)** | | | ***E. coli* strains (zone size in mm)** | |
| --- | --- | --- | --- | --- | --- | --- | --- |
|  | **S** | **R** | **N16961**  (O1 El Tor) | **BCH13298**  (O1 El Tor variant) | **Micro78**  (O1 El Tor variant) | **B2** | **IDH15978** |
| Ampicillin (10) | ≥ 17 | ≤ 13 | 6 (R) | 6 (R) | 6 (R) | 0 (R) | 0 (R) |
| Erythromycin (5) | ≥ 17 | ≤ 12 | 7 (R) | 7 (R) | 7 (R) | 6 (R) | 6 (R) |
| Chloramphenicol (5) | ≥ 18 | ≤ 12 | 6 (R) | 6 (R) | 6 (R) | 19 (S) | 19 (S) |
| Ciprofloxacin (5) | ≥ 21 | ≤ 15 | 0 (R) | 0 (R) | 0 (R) | 0 (R) | 0 (R) |
| Tetracycline (5) | ≥ 15 | ≤ 11 | 0 (R) | 0 (R) | 0 (R) | 0 (R) | 0 (R) |
| Streptomycin (10) | ≥ 17 | ≤ 12 | 0 (R) | 0 (R) | 0 (R) | 0 (R) | 0 (R) |
| Nalidixic acid (30) | ≥ 19 | ≤ 13 | 0 (R) | 0 (R) | 0 (R) | 0 (R) | 0 (R) |
| Trimethoprim / Sulfamethoxazole (1.25/23.75) | ≥ 16 | ≤ 10 | 0 (R) | 0 (R) | 0 (R) | 0 (R) | 0 (R) |
| Norfloxacin (10) | ≥ 17 | ≤ 12 | 8 (R) | 8 (R) | 8 (R) | 9 (R) | 9 (R) |
| Cefotaxime (30) | ≥ 26 | ≤ 22 | 14 (R) | 14 (R) | 14 (R) | 0 (R) | 0 (R) |
| Gentamicin (10) | ≥ 15 | ≤ 12 | 22 (S) | 22 (S) | 22 (S) | 21 (S) | 21 (S) |

| **Primers for quantitative real-time PCR** | | |
| --- | --- | --- |
| **Primers** | **Sequence (5’ – 3’)** | **Gene Locus** |
| CtxA RT FP | CCTAACAAATCCCGTCTGAGTT | VC1457 |
| CtxA RT RP | GTCTTATGCCAAGAGGACAGAG |  |
| ToxR RT FP | GACGAATAAATCGGCTCCAAAC | VC0984 |
| ToxR RT RP | AGGGTGGTTATTCGGCATATT |  |
| ToxS RT FP | GTTGTGCATCCATCTTGAACAG | VC0983 |
| ToxS RT RP | GGTTACGCCAGTCGAGTTT |  |
| TcpH RT FP | CATTGCCAGATCCTAGCTCTC | VC0827 |
| TcpH RT RP | CAACCTTTGCCGAGTTGATAAAT |  |
| TcpP RT FP | CAGCTCTGAAAGTCTAACTCAGG | VC0826 |
| TcpP RT RP | GACTACAGTCAGCTTCATCAACA |  |
| ToxT RT FP | TTACTGATGATCTTGATGCTATGGA | VC0838 |
| ToxT RT RP | ATTCTCTAAACTTTACTCCTCGAGAC |  |
| CtxB RT FP | TGTGCAGAATACCACAACAC | VC1456 |
| CtxB RT RP | TGTGAATCTATATGTTGACTACCT |  |
| TcpA RT FP | CGAAACTCTGCAGCGAATAAAG | VC0828 |
| TcpA RT RP | CGTTTCGAAATCACCAAGATCAG |  |
| RecA RT FP | GTCGCAAGCAATGCGTAAAC | VC0543 |
| RecA RT RP | CCAAACGAACAGAAGCGTAGA |  |
| VpsH RT FP | GCTACGTTAGCCCGCTATTT | VC0924 |
| VpsH RT RP | CGTGTCTCAATCACCTGTCTATC |  |
| RbmB RT FP | ATCCTGTGTACCGTGCATTT | VC0929 |
| RbmB RT RP | TCGATACCACCAGGCTCTAT |  |
| VpsR RT FP | CGAAAGTGGTACTGGGAAAGA | VC0665 |
| VpsR RT RP | TTCTGACATAGCTCGGCAATTA |  |
| VpsE RT FP | CTCCATCCTTTCGCTCTCTTG | VC0921 |
| VpsE RT RP | TTTAGGCCGCTGAGGTAAAC |  |
| AldA RT FP | CAGAGCCGAAACCATTAACAAC | VC0819 |
| AldA RT RP | CTCAAACCTGCAGAACAAACC |  |
| HlyA RT FP | CACATCACCCAGTAGCAAGT | VC0271 |
| HlyA RT RP | GCTGATTTACAGCGAAGAGAAAG |  |
| TcpB RT FP | CCTGATCGTGTCGGGTATTT | VC0829 |
| TcpB RT RP | CCAACGCCAGAGTTCTATCTT |  |
| TcpE RT FP | CTCATCTATGATCACGCCTAGC | VC0836 |
| TcpE RT RP | CTCACAGGAAGTACAGACTCAAA |  |
| AcfA RT FP | TGTGTATGTGTCACACCAACTT | VC0844 |
| AcfA RT RP | CACGAATGGAGCTCTGAGATTG |  |
| RtxC RT FP | TGATCTTCGTCGTCGTGTATTT | VC1450 |
| RtxC RT RP | CACTGCACCTTTCGGATACA |  |

**TABLE S3: List of Primers used in quantitative real-time PCR experiment, EMSA and ChIP.**

| **Primers for EMSA** | | |
| --- | --- | --- |
| **Primer** | **Sequence (5’ – 3’)** | **Amplicon**  **length (bp)** |
| EMSA TcpA FP | ATTCTCTATGTGAATGTTGCA | 150 bp |
| EMSA TcpA RP | GTCCTTTTTTAAAGAAAAAGAAA |  |
| Non-specific DNA FP | ATTGCTGAGCTAAAGGGGCTGG | 150 bp |
| Non-specific DNA RP | TGGTATTGCAGGTAGCTAGGGT |  |

| **Primers for ChIP** | | |
| --- | --- | --- |
| **Primer** | **Sequence (5’ – 3’)** | **Amplicon**  **length (bp)** |
| ChIP TcpA FP | ATTCTCTATGTGAATGTTGCA | 150 bp |
| ChIP TcpA RP | GTCCTTTTTTAAAGAAAAAGAAA |  |
| ChIP TcpI FP | ATAATTAGTTAAAAATGAAATT | 150 bp |
| ChIP TcpI RP | TTGGTTACATTATCTTTCCTGT |  |
| ChIP AcfA FP | ATTTTTACCTGTGTTTCACAT | 150 bp |
| ChIP AcfA RP | AAACAAGAATTAATTATCCTT |  |
| ChIP AcfD FP | ATTTTTACCTGTGTTTCACAT | 150 bp |
| ChIP AcfD RP | AAACAAGAATTAATTATCCTT |  |
| ChIP TagA FP | CAAAATCGTATTGAAATTTCAA | 150 bp |
| ChIP TagA RP | ATCTTACCACCACTAACTCCTC |  |
| ChIP AldA FP | AACTACAAAAAAATTACGTAAT | 150 bp |
| ChIP AldA RP | TCGATGGAAAAAACTACCTTTT |  |
| ChIP Ctx FP | TTTTACTATTTTTTCCTGATTT | 150 bp |
| ChIP Ctx RP | AATGGTATATTACGAGGGAAAC |  |

**DATA SET S1**

The FPKM values, fold change values, and P-values for all genes under two different conditions. **DATA SET S1.xlsx`**

**References**

1. Clinical and Laboratory Standards Institute (CLSI). 2015. M45-Methods for Antimicrobial Dilution and Disk Susceptibility Testing of Infrequently Isolated or Fastidious BacteriaGuidelines CLSI.
